# Factors affecting basket catheter detection of real and phantom rotors in the atria: A computational study

**DOI:** 10.1371/journal.pcbi.1006017

**Published:** 2018-03-05

**Authors:** Laura Martinez-Mateu, Lucia Romero, Ana Ferrer-Albero, Rafael Sebastian, José F. Rodríguez Matas, José Jalife, Omer Berenfeld, Javier Saiz

**Affiliations:** 1 Centro de Investigación e Innovación en Bioingeniería, Universitat Politècnica de València, Valencia, Spain; 2 Computational Multiscale Simulation Lab, Department of Computer Science, Universitat de València, Valencia, Spain; 3 Dipartimento di Chimica, Materiali e Ingegneria Chimica “Giulio Natta”, Politecnico di Milano, Milano, Italy; 4 Center for Arrhythmia Research, University of Michigan, Ann Arbor, Michigan, United States of America; 5 Fundación Centro Nacional de Investigaciones Cardiovasculares (CNIC), Madrid, Spain; 6 CIBER of Cardiovascular Diseases, Madrid, Spain; University of California San Diego, UNITED STATES

## Abstract

Anatomically based procedures to ablate atrial fibrillation (AF) are often successful in terminating paroxysmal AF. However, the ability to terminate persistent AF remains disappointing. New mechanistic approaches use multiple-electrode basket catheter mapping to localize and target AF drivers in the form of rotors but significant concerns remain about their accuracy. We aimed to evaluate how electrode-endocardium distance, far-field sources and inter-electrode distance affect the accuracy of localizing rotors. Sustained rotor activation of the atria was simulated numerically and mapped using a virtual basket catheter with varying electrode densities placed at different positions within the atrial cavity. Unipolar electrograms were calculated on the entire endocardial surface and at each of the electrodes. Rotors were tracked on the interpolated basket phase maps and compared with the respective atrial voltage and endocardial phase maps, which served as references. Rotor detection by the basket maps varied between 35–94% of the simulation time, depending on the basket’s position and the electrode-to-endocardial wall distance. However, two different types of phantom rotors appeared also on the basket maps. The first type was due to the far-field sources and the second type was due to interpolation between the electrodes; increasing electrode density decreased the incidence of the second but not the first type of phantom rotors. In the simulations study, basket catheter-based phase mapping detected rotors even when the basket was not in full contact with the endocardial wall, but always generated a number of phantom rotors in the presence of only a single real rotor, which would be the desired ablation target. Phantom rotors may mislead and contribute to failure in AF ablation procedures.

## Author summary

In computer simulations we determined the accuracy of using multiple-electrode basket catheters to detect atrial fibrillation sources (rotors) during an ablation procedure. We used a realistic 3D atrial model and a virtual multiple-electrode basket catheter placed in three different positions inside the right atrium. The ability to detect a true rotor depended on three major factors: 1) the position of the basket inside the atrium, 2) the distance between the electrodes and the atrial wall, and 3) the inter-electrode distance. When the electrodes were not in full contact with the atrial wall far-field sources predominated on the recorded signal. As a consequence, phantom rotors were generated on the maps. Interpolation of the signals increased the false rotor incidence, whereas increasing the electrode density decreased it. We conclude that the appearance of false rotors might contribute to AF ablation procedure failure because the physician may erroneously target atrial tissue locations where no true rotor exists.

## Introduction

Atrial fibrillation (AF) is the most common cardiac arrhythmia seen in clinical practice and is the most important cause of embolic stroke[1,2]. Recently catheter ablation has been recommended as a first-line treatment for AF termination[3]. Traditionally ablation procedures aimed at terminating AF have been primarily focused on isolating the pulmonary veins (PVs)[4–6] often complemented by linear ablation of the posterior left atrium (LA)[7]. Recent approaches based on mapping the electrical activity during AF take into account the underlying mechanism and target the AF drivers[8,9], as has been demonstrated by high-resolution optical mapping of AF in animal models[10–13] and explanted human hearts[14].

Unfortunately, clinical mapping approaches are limited to the use of low resolution multi-electrode systems (contact and non-contact)[8,15–21], but the use of panoramic contact multi-electrode basket catheters to map the atria in search for AF drivers enabled >80% success rates in some studies compared to 20–50% obtained by conventional ablation[1,15,22]. However, whether rotors are AF drivers remains controversial[18,19,23,24] and the use of multi-electrode mapping approaches to target rotors needs further studies to validate their accuracy and applicability in the clinic.

We surmised that several factors may limit the accuracy of basket catheter-based phase mapping in localizing rotors, including: (1) the electrode-endocardium distance; (2) the effects of distant electrical sources and (3) inter-electrode interpolation. Here we use computer simulations to analyze how those factors affect the accuracy of basket catheters in mapping AF-like electrical activity and detecting rotors.

## Materials and methods

### Geometrical and electrical atrial model

We used a recently developed realistic 3D model of the human atria[25] that included heterogeneity at cell, tissue and organ scale. It comprises the following anatomical regions with their respective fiber orientation: right atrium (RA), left atrium (LA), crista terminalis (CT), fossa ovalis (FO), pulmonary veins (PV), Bachman’s bundle (BB), divided into left (BBLA) and right (BBRA) sides, pectinate muscles (PM), isthmus (IST), sinoatrial node (SAN), coronary sinus (CS), mitral valve ring (MVR), tricuspid valve ring (TVR), right atrial appendage (RAA), left atrial appendage (LAA), superior vena cava (SVC) and inferior vena cava (IVC). This computational finite element model is composed of linear hexahedral elements with a regular spatial resolution of 300 μm, and wall thickness between 600 and 900 μm (754893 nodes and 515010 elements). See [25] for additional details.

**Fig 1:**
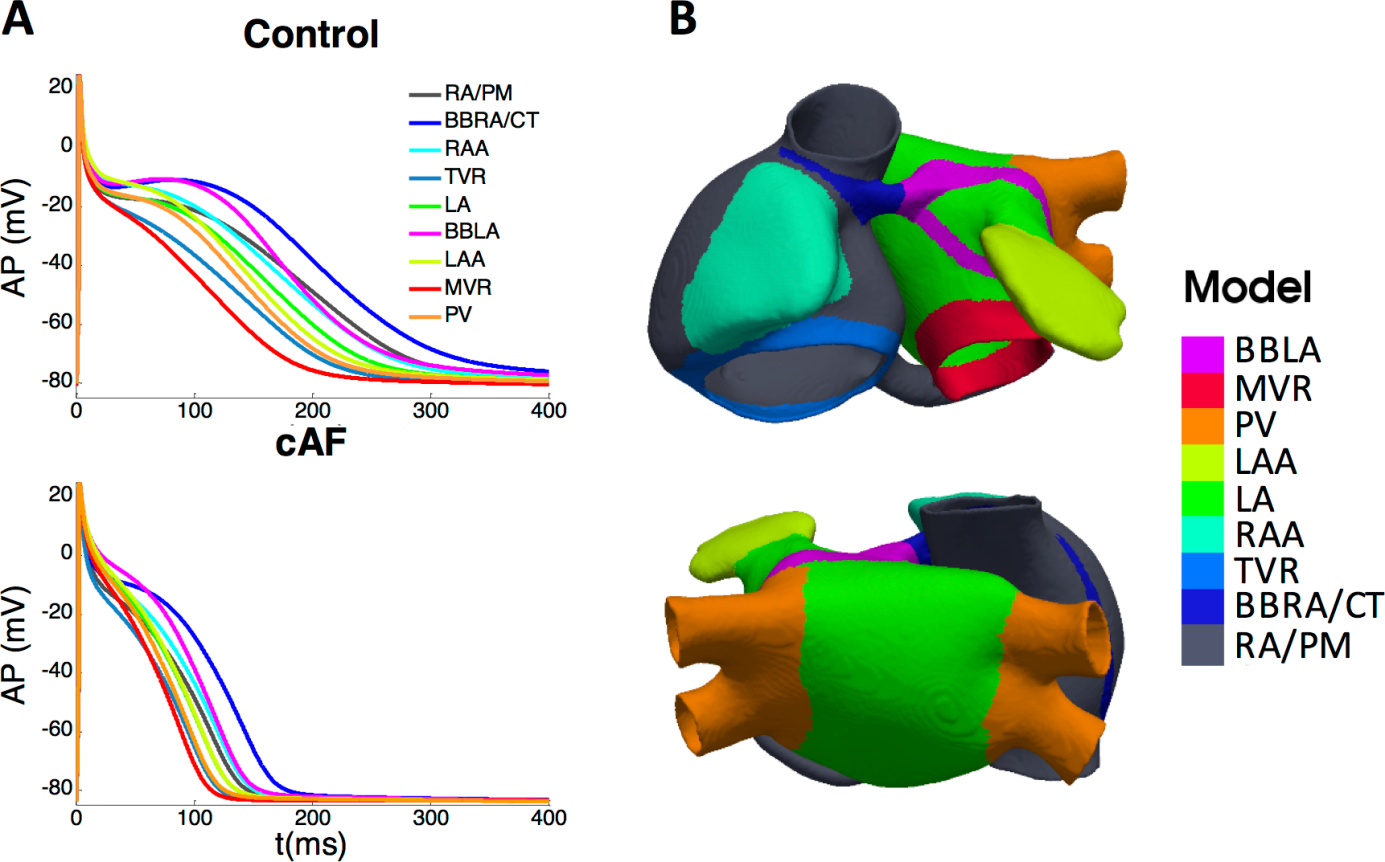
Action potentials (APs) and their corresponding atrial regions. A) AP produced by each variation of the CRN model in control (top) and chronic AF (bottom) conditions. B) Atrial regions to which each cell model was assigned.

The electrical activity in our model was solved by the monodomain formalism, and the cellular ion kinetics by the Courtemanche-Ramírez-Nattel ionic model[26]. In order to reproduce transmembrane potential (V_m_) of experimentally observed heterogeneity in action potentials (APs) morphology and duration in different regions of the atria[27–31], the maximum conductance of three ionic currents (I_to_, I_CaL_ and I_Kr_) was adjusted as described previously[25,32–35] (see Table 1). This procedure yielded nine cellular models (RA/PM, LA, CT/BBRA, BBLA, PV, MVR, TVR, RAA and LAA) whose APs are depicted in Fig 1A (top); AP durations (APD) to 90% and 95% repolarization (APD_90_ and APD_95_) are shown in Table 1. APs were recorded after 1 minute of stimulation at a basic cycle length (BCL) of 1000 ms; pulse amplitude and duration were 28 pA/pF and 2 ms, respectively. The APD variation among regions was similar to experimental observations[27–29]: APD was longer in the CT region than in the RA region, where it was longer than in the TVR, RAA and LA regions; and APD was shorter in the PV region than in the LA region.

**Table 1:** Variation in the ionic channel conductances used to reproduce the atrial heterogeneity and the APD distribution under control conditions.

	Atrial region								
	RA/PM	CT/BBRA	TVR	RAA	LA	BBLA	MVR	LAA	PV
${g_{to}}_{max}$^**a**^	1.00	1.00	1.00	0.68	1.00	1.00	1.00	0.68	1.00
${g_{CaL}}_{max}$^**a**^	1.00	1.67	0.67	1.00	1.00	1.67	0.67	1.00	1.00
${g_{Kr}}_{max}$^**a**^	1.00	1.00	1.53	1.00	1.60	1.60	2.44	1.60	2.20
**APD_95_ (ms)^b^**	339	375	264	325	299	324	228	281	268
**ΔAPD_95_ (%)^c^**	0	10	-22	-4					
**Exp. ΔAPD_95_ (%)^d^**		42[27]	-15[27]	-5[27]					
**APD_90_ (ms)^e^**	295	311	227	278	256	261	193	236	227
**ΔAPD_90_ (%)^f^**					-13				-11
**Exp. ΔAPD_90_ (%)^g^**					-10[28]				-11[29]

Variations of the channel’s conductance (third to fifth rows) in each region (second row, second to tenth column) used to reproduce the atrial heterogeneity; APD_90_ and APD_95_ obtained in each atrial region and their respective increments (sixth, seventh, ninth and tenth rows); and comparison with the experimental variation of APD between regions (eighth and eleventh rows).

^a^Relative values with respect to the g_max_ in the original CRN model[26]

^b^APD_95_ and

^e^APD_90_ after 1 minute of stimulation at BCL = 1000ms

^c^Variation of the APD_95_ with respect to the APD_95_ in the RA/PM region

^d,g^Experimental values for ΔAPD_95_ and ΔAPD_90_, respectively

^f^Variation of the APD_90_ in LA with respect to the APD_90_ in RA/PM and in PV with respect to the LA

Then the nine cellular models corresponding to healthy conditions were assigned to the nodes in the 3D geometrical model, following the distribution shown in Fig 1B. Tissue conductivities in each region were tuned as in[25] to match the activation sequences to experimental data[36] (see S1 Fig).

Atrial electrical remodeling corresponding to chronic AF (cAF) was also introduced through the variation of the maximum conductances of I_to_, I_CaL_, I_K1_, I_Kur_ and I_Ks_ (see Table 2)[37–42], similarly to other computational studies[33,43–47]. Remodeling in the RA (RA, CT/BBRA, TVR and RAA regional models) was different to that in the LA (LA, BBLA, MVR, LAA and PV regional models), according to experimental data reported in the literature (resulting APs depicted in Fig 1A bottom). APD shortening was within the range of the available experimental observations[38,40] (Table 3). The maximum dispersion of APD_90_ due to heterogeneity in our model was 57 ms in cAF, compared to 125 ms under control conditions.

**Table 2:** Variations in the ionic channel currents used to reproduce the electrical remodeling experimentally observed under cAF conditions.

cAF	RA	LA	References
**g_to_**	-45%	-75%	Caballero et al[37]
**g_CaL_**	-65%	-65%	Van Wagoner et al[39], Workman et al[40]
**g_K1_**	+100%	+100%	Dobrev et al[41], Voigt et al[42], Bosch et al[38]
**g_Kur_**	-60%	-45%	Caballero et al[37]
**g_Ks_**	+150%	+100%	Caballero et al[37]

The maximum conductance of several ionic channels was modified differently in the RA than in the LA, accordingly to experimental works reported in the literature.

**Table 3:** APD_90_ reductions after the electrical remodeling.

	RA/PM	CT/BBRA	TVR	RAA	LA	BBLA	MVR	LAA	PV	Exp. Data
**APD**_**90-Ctrl**_ **(ms)**^**a**^	266	295	197	252	230	253	170	216	204	193 [40] / 218 [38]
**APD**_**90-cAF**_ **(ms)**^**a**^	127	156	106	133	119	135	99	119	109	93 [40] / 100 [38]
**ΔAPD**_**90**_ **(%)**	-52	-47	-46	-47	-48	-46	-42	-45	-47	-52 [40] / -54 [38]

APD_90-Ctrl_: APD at 90% repolarization in control conditions; APD_90-cAF_: APD at 90% repolarization in cAF conditions; ΔAPD_90_: difference of APD_90_ between cAF and control conditions in each atrial region; Exp. Data: experimental data.

^a^APD_90_ after 1 minute of stimulation at BCL = 500 ms

The nine remodeled cellular models were considered across the atria as for control conditions (Fig 1B), with 15% reduced intracellular conductivity to account for gap junctional remodeling[48,49] as in other simulation studies[44,47]. Then we applied 21 stimuli (BCL 500 ms, amplitude 28 pA/pF, duration 2 ms) to the SAN region to stabilize models in neighboring regions. The electrical and gap junctional remodeling produced a reduction of 17% in the conduction velocity with respect to control, consistent with experimental observations[49]. To generate reentrant activity, after the 21 stabilization pulses, we paced the CS using a continuous high frequency train of ectopic foci (cycle length = 110 ms) and the simulation was run for an additional 12 seconds, in which the simulated activity was driven by 2 stable sources: the pacing train near the CS and a stable rotor near the CT.

Stimulation from the SAN was stopped upon starting the ectopic pacing from the CS because, in test simulations, when SAN stimulation was maintained, its discharge propagation was overridden by the faster ectopy rate (110 ms vs 500 ms) and differences in the propagation patterns were negligible.

### EGMs calculation and phase maps

Unipolar EGMs inside the atrial cavity and on its endocardial boundaries were computed as extracellular potentials, with a temporal resolution of 1 ms, in a whole atrial-torso model developed previously[25]. The torso model developed in[25] was re-meshed in order to improve the spatial resolution at: 1) the endocardium-blood interface to enhance the accuracy when detecting the rotor’s tip trajectory; 2) the atrial blood to introduce the EGMs at the basket electrodes in several positions. Accordingly, the resulting torso mesh had 254976 nodes and 1554255 tetrahedral elements with a spatial resolution ranging approximately from 0.5 mm on the atrial endocardium-blood interface to 5.8 mm on the torso surface. The number of nodes belonging to the endocardium-blood interface were 25175 in the RA and 24819 in the LA.

The extracellular potentials were computed by the bidomain formalism in two steps[50]. By assuming equal anisotropy ratios for the intracellular, D_i_, and extracellular, D_e_, conductance tensors (D_e_ = λD_i_), the bidomain equations can be decoupled into an equation describing the changes in the transmembrane potential, V_m_, and another equation describing the extracellular potential, V_e_, in the heart domain[51]:

$$\nabla ∙(D\nabla V_{m})=C_{m}∙\frac{\partial V_{m}}{\partial t}+I_{ion\;}in\;\Omega_{H}$$ (1)

$$\nabla ∙(D\nabla V_{e})=-\frac{1}{1+\lambda }\nabla ∙(D\nabla V_{m})\;in\;\Omega_{H},$$ (2)

where $D=\frac{\lambda }{1+\lambda }D_{i}$ is the equivalent conductivity tensor, V_m_ is the transmembrane potential, V_e_ is the extracellular potential, I_ion_ is the transmembrane ionic current that depends on the cellular model, C_m_ is the membrane capacitance and Ω_H_ is the heart domain. Eqs (1) and (2) are subjected to the following boundary conditions in Ω_H_:

$$n∙(D\nabla V_{m})=0\;on\;{\partial \Omega }_{H}$$ (3)

$$n∙(D\nabla V_{e})=0\;on\;{\partial \Omega }_{H},$$ (4)

where n is the outward normal to ∂Ω_H_. Eqs (1) and (3) allow for solving the V_m_ in the cardiac tissue, whereas Eq (2) and (4) recast the V_e_ in the heart tissue after V_m_ has been calculated. Note that boundary conditions (3) and (4) consider the heart to be immersed in a non-conducting bath.

To calculate the EGMs inside the heart cavity and on the basket electrode points, we need to place the heart within the torso and solve for the extracellular potential in the entire domain (the heart Ω_H_ and the torso Ω_T_ outside of the heart). Therefore, we define our 3D space problem to include the governing equations for the solid conductor associated with the torso, and modify accordingly the boundary conditions at the heart/torso interface, i.e., ∂Ω_H_. Under the hypothesis of equal anisotropy ratio for D_i_ and D_e_, the extracellular potential in the domain Ω_H_∪Ω_T_, after obtaining V_m_ as solution of Eq (1) and (3), is found as the solution of the following Laplace Equation:

$$\nabla ∙(D_{T}\nabla V_{T})=0\;in\;\Omega_{T}$$ (5)

where V_T_ and D_T_ are the extracellular potential and heterogeneous conductance tensor in the torso, respectively. Eq (5) is subjected to the following boundary and continuity conditions:

$$V_{e}=V_{T}\;\mathrm{in}\;\partial \Omega_{H}$$ (6)

$$n∙(D\nabla V_{T})=0\;in\;{\partial \Omega }_{T}$$ (7)

where ∂Ω_T_ is the torso-air non-flux boundary. Then, V_T_ is the EGM at either the basket electrodes positions or at the endocardial surface.

Next, endocardial and cavity EGMs (i.e, the V_T_) were bandpass filtered (7–10 Hz) in order to allow rotor tip tracking similarly to[52]. Phase maps were calculated from the EGMs during the last 11 s of the simulation, since the reentrant activity during the first second was not stable. In the custom-made software routines implemented in MATLAB (MathWorks, Natick, MA), we applied the Hilbert transform (HT) to the filtered EGMs (EGM_f_(t), Eq 8), as in prior studies[11,53,54] and computed the instantaneous phase θ, whose values ranged from -π to π radians (Eq 9).

$$HT[{EGM}_{f}(t)]=\frac{1}{\pi }\cdot \int_{-\infty }^{\infty }\frac{{EGM}_{f}(\tau )}{t-\tau }\cdot d\tau$$ (8)

$$\theta =\tan^{-1}(\frac{\mathrm{HT}[{EGM}_{f}(t)]}{{EGM}_{f}(t)}).$$ (9)

We excluded the first and last 500 ms of the signals to avoid filtering and transformation artefacts, resulting in phase maps as frames of movies of 10-s long. Finally the phase singularity points (PSs), where all phases converge, were computed to track the rotor’s trajectory on the endocardium-blood interface [55–58] when:

∮∇θ·dr=±2π (10)

where r is the closed curve surrounding the singularity point at the center of the reentry. To approximate Eq (10) and automatically localize PSs, we adapted the method proposed by Rogers[59]. Accordingly, the endocardium-blood interface corresponds to a 3D surface mesh of triangular elements and each node in the mesh has a phase value. For each element in the mesh and for all simulation time steps we computed the spatial variations of phase among its nodes (space gradients). For those elements around which all phases converged, the summed phase variation along a closed surrounding path was ±2π. Those elements were designated as PSs and painted in white superimposed on the phase maps (see S1 Video panel B). The automatic PS detection algorithm was implemented in MATLAB. A similar procedure was performed on the basket phase maps. The closed path integral was computed along the edges of each triangular element (with a perimeter of approximately 0.5 mm in case of the 2D projections of the basket, and 2 mm in case of the endocardial-blood surface). Rotors were defined as an excitatory wave pivoting around a PS for at least one cycle of rotation and were visually detected in phase map movies (see S1–S4 Videos).

### The basket catheter

**Fig 2:**
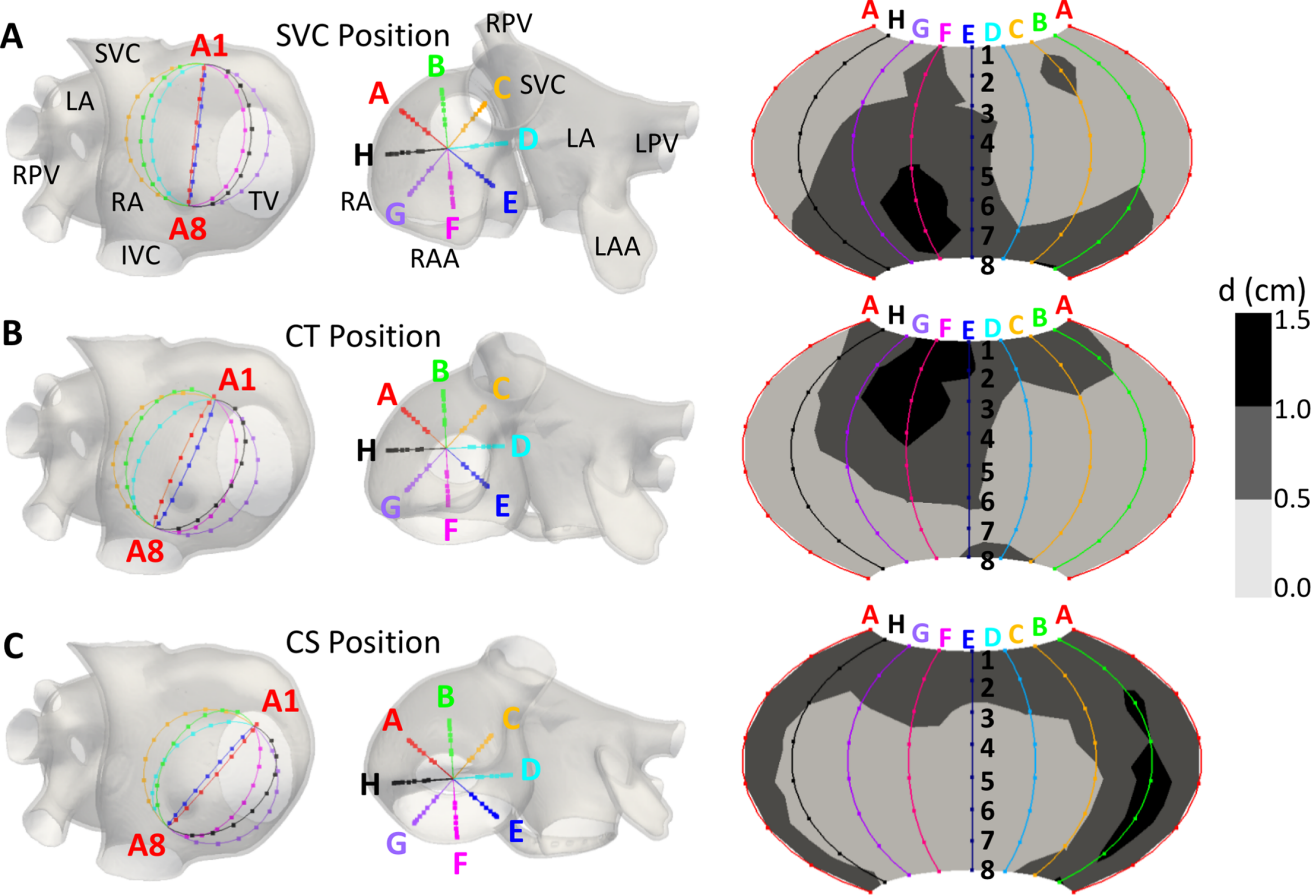
Positions of the 64-pole basket mapping catheter in the RA. A-C) SVC, CT and CS position (first and second column) and electrode-endocardium distance maps for the 64 electrodes in each position (third column). SVC and IVC: superior and inferior vena cava; LA and RA: left and right atrium; LPV and RPV: left and right pulmonary veins; TV: tricuspid valve; LAA and RAA: left and right atrial appendage.

A virtual intracardiac 64-pole mapping basket catheter formed by 8 splines (A-H), each containing 8 electrodes (1–8), was placed in three different positions in the RA of our atrial model: close to the SVC, CT and CS (Fig 2). The diameter of the 8×8-electrode basket modelled was 31 mm, corresponding to the smallest Constellation mapping catheter (Boston Scientific). Electrodes were equidistant along the splines with an inter-electrode distance of 4.8 mm. The distances between electrodes at neighboring splines varied between 5.4 mm for the electrodes near the poles and 11.7 mm for the electrodes near the equator (see S2A Fig).

EGMs were computed at the 64 electrodes’ coordinates and linearly interpolated on 57600 points on a periodic 2D projection of the basket (Fig 2A–2C right column). The same signal processing explained above was applied to the basket’s EGMs, in order to obtain the phase maps and PSs detection.

The distribution of the distance (d) between each electrode in the basket and the closest point on the endocardial surface differed for the three basket positions within the atria, but the number of electrodes located at d≤0.5 cm and 0.5<d≤1 cm was approximately similar (Fig 2A–2C right column). In the first case the percentage of electrodes was 53%, 58% and 50%, and in the second case 41%, 34% and 42%, for the SCV, CT and CS positions, respectively.

Finally, to analyze the effect of electrode density, we varied the number of electrodes in the basket between 4×6 and 16×16 for each of the three positions (see S2 Fig).

### Numerical and computational methods

The mono-domain formulation (Eqs 1–4) was solved using the operator splitting numerical scheme with ELVIRA software[60] with a constant time step dt = 0.01 ms. Simulation of 12 s of atrial activity took 37 hours on a computing node with eight 6-core AMD Opteron Processors 6234 clocked at 2.4 GHz. The approximation of the bidomain formulation (Eqs 5–7), the phase maps (Eqs 8–9) and the PSs detection (Eq 10) were computed with custom-made software routines in MATLAB (MathWorks, Natick, MA).

## Results

The goal of this simulation study was to evaluate how each of the following three factors: (1) electrode-endocardium distance, (2) distant sources and (3) inter-electrode interpolation, affected the detection of rotors when using a basket mapping catheter in the atria. To accomplish that goal, the basket maps were compared to the activation patterns of AP simulated on the atrial wall, which serve as our ground-true reference.

### The simulated propagation patterns

**Fig 3:**
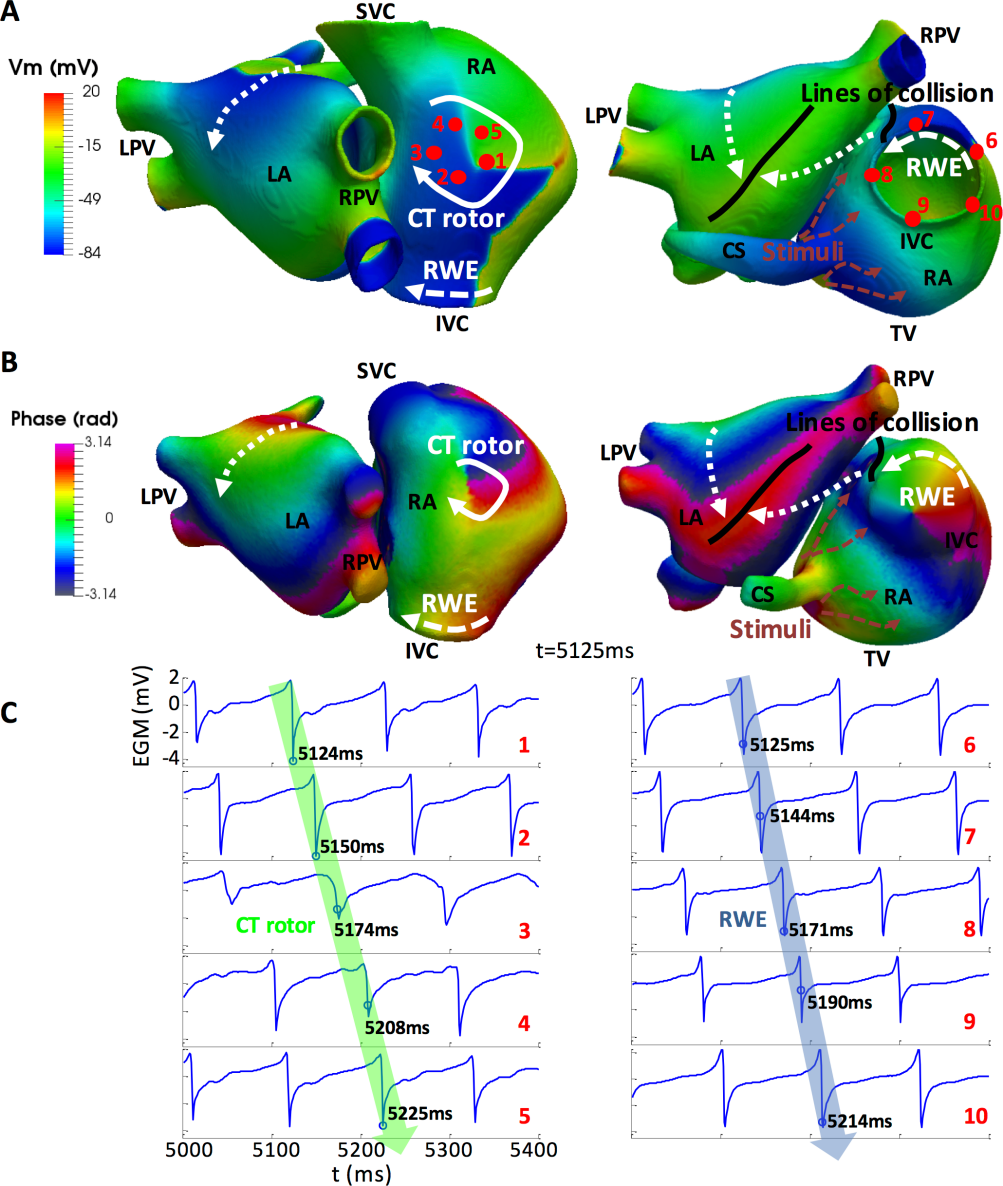
Electrical propagation and phase maps. A) Snapshot of the simulated transmembrane potential (V_m_) in the 3D atrial model at t = 5125 ms. CT rotor (white arrow), RWE (dashed white arrow), CS stimuli wave front (orange dashed arrows), wave fronts in the LA (dotted white arrows) and lines of collision (black lines). B) Snapshot of the EGMs phase maps, computed on the endocardium-blood interface after filtering, at t = 5125 ms. C) EGM traces corresponding to points 1–10 in panel A. Blue circles on the traces show the closest activation times to t = 5125 ms. Green and blue arrows superimposed over the EGMs demonstrate the sequential activation times of the tissue at points 1–5 (CT rotor) and 6–10 (RWE), respectively. SVC and IVC: superior and inferior vena cava; RPV and LPV: right and left pulmonary veins; RA and LA: right and left atrium; TV: tricuspid valve; CS: coronary sinus; CT rotor: rotor on the crista terminalis; RWE: rotor wave extension.

Application of a sustained high frequency train of stimuli to the RA close to the CS led to a complex propagation pattern maintained by a stable rotor on the crista terminalis (CT rotor) accompanied by a distal rotor wave extension (RWE) reentry around the inferior vena cava (IVC; Panel A in S1 Video). The CT rotor migrated back and forth between the superior vena cava (SVC) and the IVC along the CT, while its RWE persistently collided and merged with the wavefront generated by the CS stimuli. Propagation in the LA was more regular. The LA was repeatedly excited by two wave fronts from the RA: one moving toward the roof through Bachmann´s bundle (BB) and spreading to the posterior wall and the other moving toward the inferior wall through the foramen ovale (FO) limb and extending to the posterior wall. Both wave fronts collided between the posterior and inferior walls, with the precise location of the collision line varying throughout the simulation. Fig 3A is a snapshot at 5125 ms showing the CT rotor (white arrow), the RWE (dashed white arrow), the direction of the propagation in the LA (dotted white arrows), the wave front corresponding to the high frequency train of stimuli applied near the CS (dashed orange arrows), the collision between the CS stimuli wave front and the RWE (black line close to the FO) and the collision in the LA (black line between the posterior and inferior walls).

The EGMs and their corresponding phase maps were calculated at the endocardium-blood interface. The trajectory described by the CT rotor tip, detected through the PSs calculations, is depicted in white superimposed on the phase maps (Panel B in S1 Video). All the events described above for the propagation maps were also identified on the phase maps, as shown in Fig 3B. Furthermore, not only the CT rotor but also the RWE circulating around the IVC appeared as a rotor whose tip was in the middle of the orifice. The rotational activation of the tissue can be observed in the EGMs shown in Fig 3C. The EGMs on the left correspond to red points 1–5 (CT rotor) in Fig 3A, while the EGMs on the right correspond to red points 6–10 (RWE). Their corresponding phases are shown in S3A Fig.

### Effect of electrode-endocardium distance on mapping

**Fig 4:**
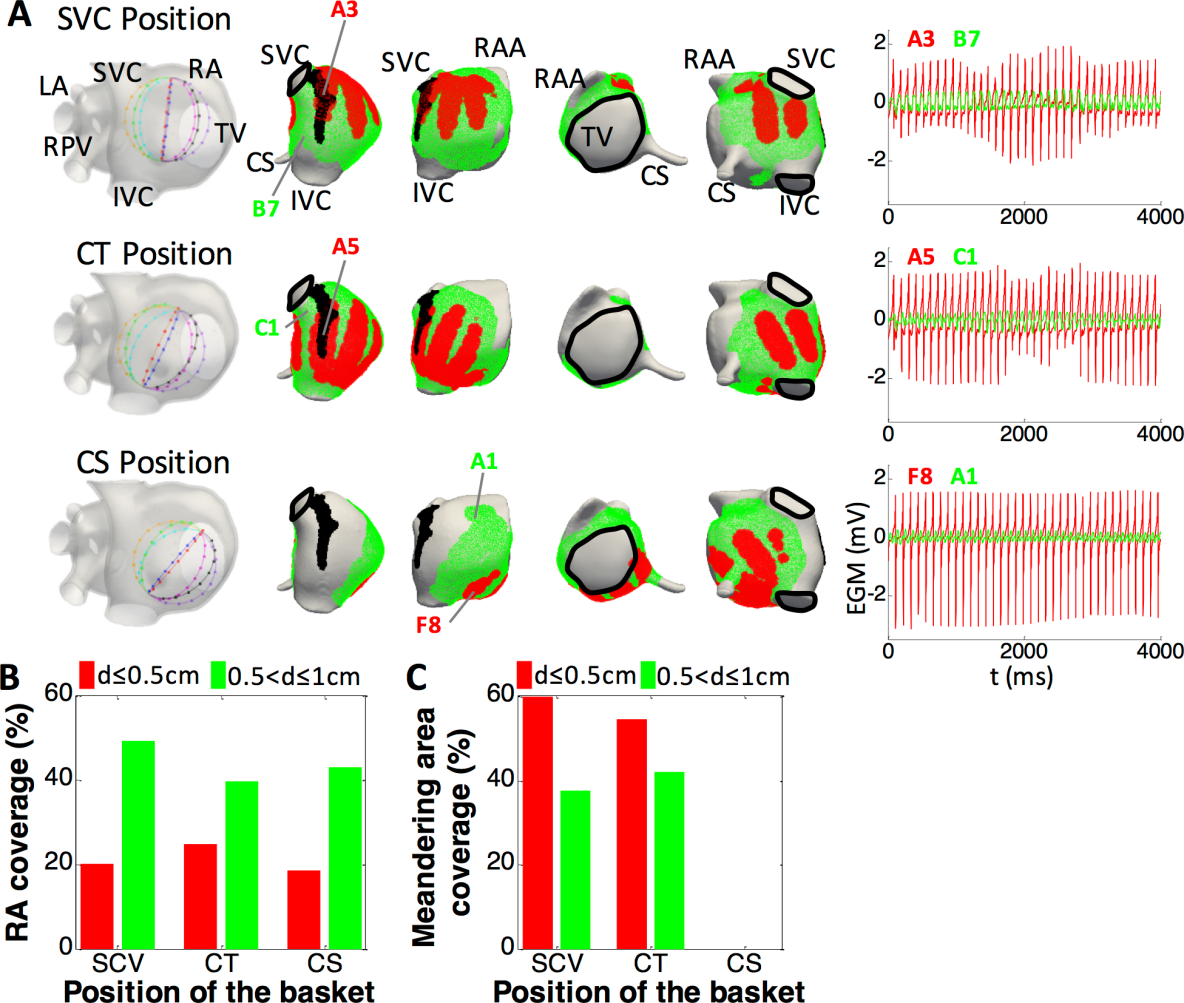
RA and the CT rotor meandering area in relationship with the near and far basket electrodes in the SVC, CT and CS positions. A) Meandering area of the CT rotor (black) and region of the endocardium located at a distance d ≤ 0.5 cm (red) and 0.5 < d ≤ 1.0 cm (green) from at least one electrode for the SVC (top), CT (middle) and CS (bottom) positions. Panels illustrating the positions of the basket inside the RA have been also included on the left side for clarity. Right side column depicts EGM traces from electrodes set at d ≤ 0.5 cm (red) and 0.5 < d ≤ 1.0 cm (green) for the SVC, CT and CS positions. B) RA coverage by the basket computed as the ratio between the section of the endocardium at a distance d ≤ 0.5 cm (red) and 0.5 < d ≤ 1.0 cm (green) from at least one electrode over the whole interface for the three basket positions. C) Coverage of the CT rotor meandering area computed as the ratio between the section of the meandering area overlapped to the RA coverage over the whole meandering area. SVC and IVC: superior and inferior vena cava; LA and RA: left and right atrium; RPV: right pulmonary veins; TVR: tricuspid valve ring; CS: coronary sinus; RAA: right atrial appendage.

The amplitude of the EGMs depends on the dipole strength and the source-to-electrode distance (see Fig 4). For all three different basket positions (SVC, CT and CS) we analyzed the effect of the electrode-endocardium distance (d) at each electrode location by computing EGMs for all 64 electrode coordinates to build phase maps. We also evaluated the RA and the meandering area coverages for each basket position. First, the RA coverage was defined as the percentage of endocardium at a distance d ≤ 0.5 cm (red) or 0.5 < d ≤ 1.0 cm (green) from at least one electrode of the basket (Fig 4A) over the whole atrial tissue. RA coverage was similar for the three basket positions (~20% for d ≤ 0.5 cm and ~45% for 0.5 < d ≤ 1.0 cm, as shown in Fig 4B). Second, the meandering area coverage was defined as the percentage of CT rotor meandering area (black area in panel A, corresponding to the region of the endocardium comprising the rotor trajectory) superimposed on the RA coverage (red or green area in panel A) over the whole CT rotor meandering area. Meandering area coverage was ~60% for d ≤ 0.5 cm and ~40% for 0.5 < d ≤ 1.0 cm for the SVC and CT positions (Fig 4A, top and middle, and Fig 4C), while it remained completely uncovered for the CS position (Fig 4A, bottom, and Fig 4C). As an example of the variation of amplitude regarding the distance to the tissue, the traces on the right-hand side in panel A of Fig 4 shows EGMs on electrodes set at d ≤ 0.5 cm (Red traces; A3, A5 and F8 for the SVC, CT and CS positions, respectively) and at 0.5 < d ≤ 1.0 cm (Green traces; B7, C1 and A1 for the SVC, CT and CS positions, respectively). The maximum root mean square value (V_rms_) was 0.74 mV among all the EGMs corresponding to the electrodes set at d ≤ 0.5 cm, while it was 0.33 mV for the electrodes set at 0.5 < d ≤ 1.0 cm. Interestingly, a relatively large variation on the nearest EGMs amplitude at electrodes A3 and A5 is observed in the SVC and CT position which could be related to rotor meandering.

**Fig 5:**
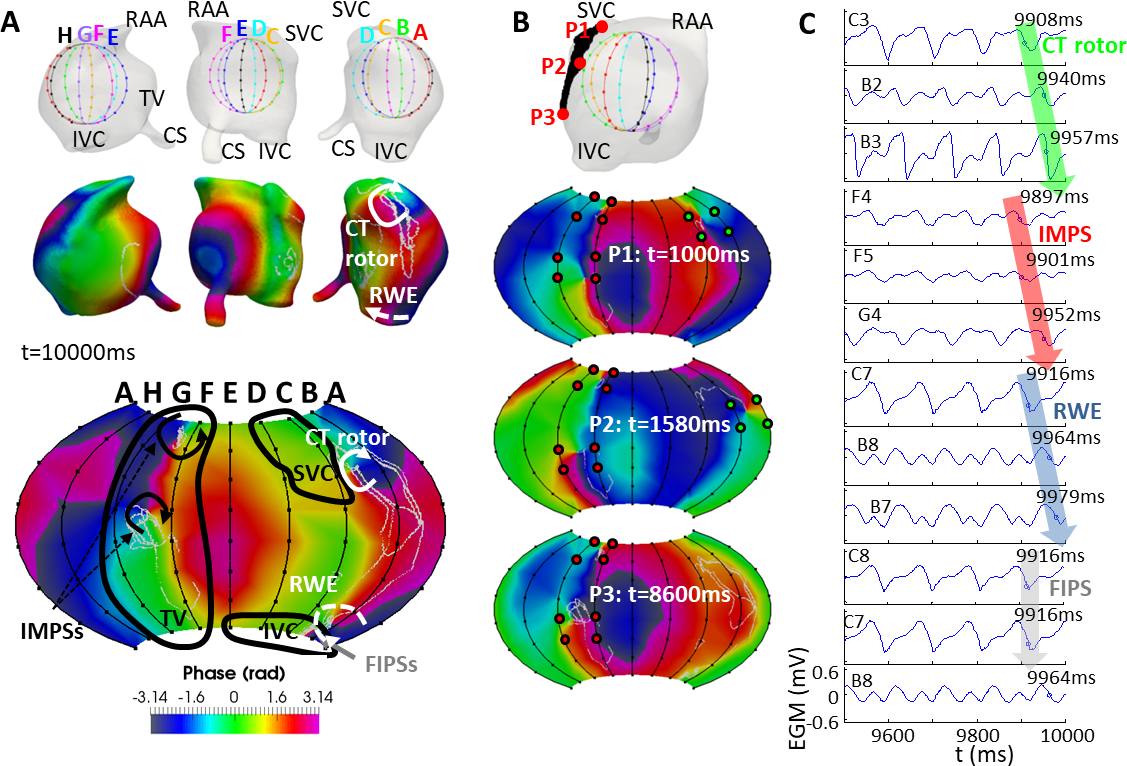
Basket mapping for the SVC position. A) Views of the location of the basket in the RA (top row), snapshots of the phase maps in the endocardium-blood interface (middle row) and of the phase map computed from the basket’s recordings (bottom row) at t = 10000 ms. PSs of the whole simulation were depicted in white over the phase maps (middle and bottom row). The black curves on the basket’s phase map correspond to the SVC, IVC and TVR orifices. CT rotor (white arrow): rotor on the crista terminalis; RWE (white dashed arrow): rotor wave extension; IMPSs (black arrows): imaginary phase singularities; FIPSs (grey arrow): false interpolation phase singularities. B) Selection of points (P1, P2 and P3) belonging to the CT rotor meandering area (top row); snapshots of the basket phase map when the CT rotor was moving through the points P1-P3 (second to bottom row). Green points denote the electrodes surrounding the CT rotor and red points denote the electrodes surrounding the IMPSs. C) EGMs at electrodes detecting the CT rotor (C3, B2, B3), IMPSs (F4, F5, G4), RWE (C7, B8, B7) and FIPS (C8, C7, B8). Blue circles on the plot of the EGMs indicate the activation time closest to t = 10000 ms. Green, red and blue arrows over the EGMs enhance the sequential activation of the electrodes detecting the CT rotor, IMPSs and RWE, respectively. However, the grey arrow over the EGMs regarding the FIPS shows the non-sequential activation of the electrodes. SVC and IVC: superior and inferior vena cava; RAA: right atrial appendage; TV: tricuspid valve; CS: coronary sinus; CT rotor: rotor on the crista terminalis; RWE: rotor wave extension. IMPS: Imaginary phase singularity; FIPS: false interpolation phase singularity.

**Fig 6:**
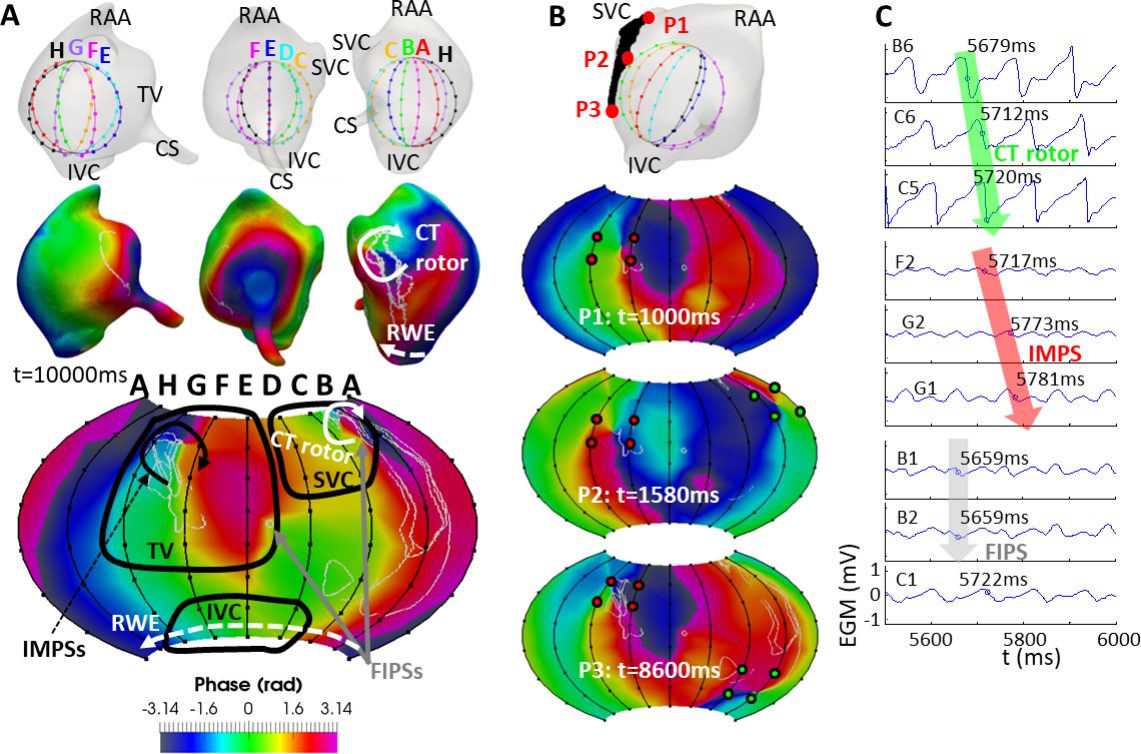
Basket mapping for the CT position. The layout of this Fig is the same as in Fig 5.

**Fig 7:**
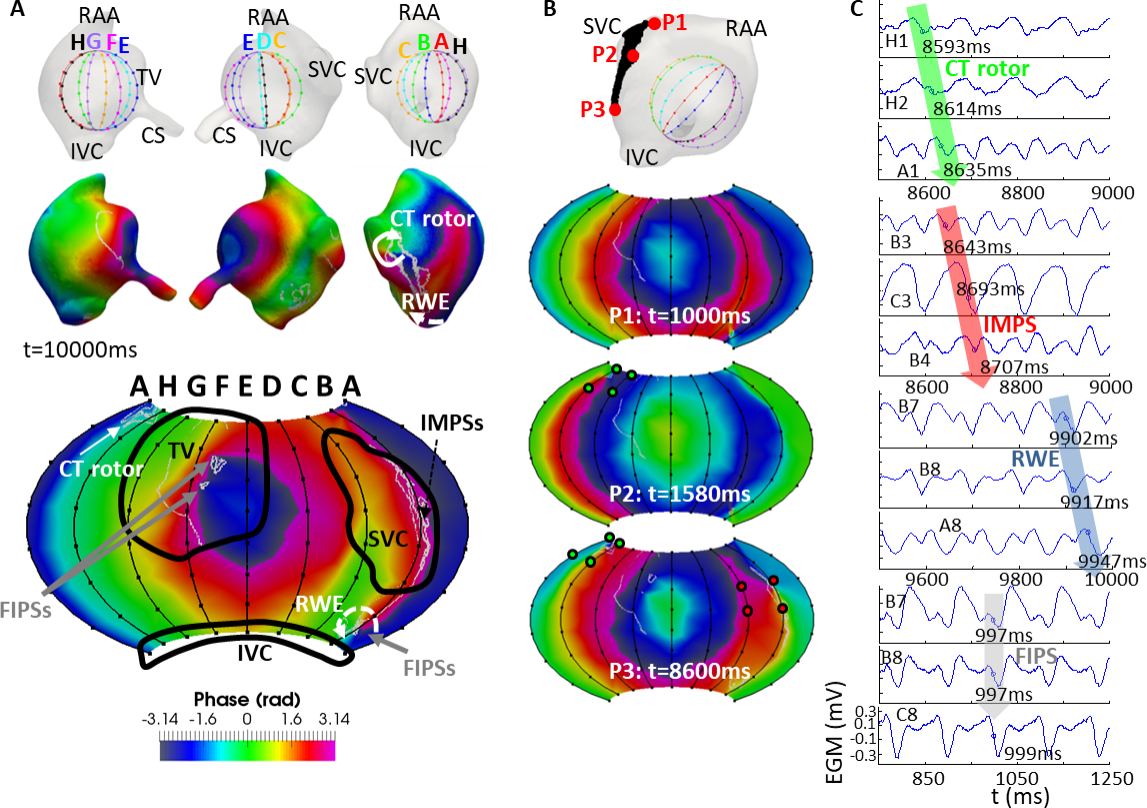
Basket mapping for the CS position. The layout of this Fig is the same as in Fig 5.

The effects of the 3 different positioning of the basket within the RA on the characteristics of its rotor mapping are illustrated in Figs 5–7. Surprisingly, in addition to the CT with its RWE on the atrial surface (see Fig 3), the basket phase maps also show phantom PSs at various locations that have no corresponding reentrant AP on the atria. We classified the false PSs either as an imaginary phase singularity (IMPS), when activation occurred sequentially in the surrounding electrodes, or as a false interpolation phase singularity (FIPS), when the electrodes surrounding the singularity did not register the activation sequentially. FIPSs appear because of the inter-electrode interpolation of the EGMs prior to the computation of the phase maps (see below). For the three basket positions tested, a comparison between the PSs locations on the phase maps and the electrode-to-endocardial surface distance maps (Figs 2 and 4) shows that IMPSs and FIPSs appear at various distances, including d≤0.5 cm, suggesting that near- and far-field sources have an influence on the generation of PSs.

Starting from the SVC position in Fig 5, panel A illustrates the location of the basket at the high RA (top row), the phase maps on the endocardium-blood interface (middle row) and the phase map corresponding to the basket recordings (bottom) at t = 10000 ms. White colored PS trajectories are superimposed on the phase maps. The basket phase map was visually more like the endocardium-blood interface map at splines A-B-C-D than at splines E-F-G-H, where a higher number of electrodes were at d>0.5 cm from the tissue (see Figs 2 and 4). The CT rotor was detected 94% of the simulation time at splines A-B-C (white clockwise arrow in Fig 5A). The CT rotor was not detected when it migrated closer to the IVC (6% of the simulation time). A counter-rotating wave to the CT rotor was found to rotate around the IVC and is termed in this study as a “rotor wave extension” (RWE). The basket map detected the RWE mostly uninterruptedly around the IVC, near the basket’s south pole (dashed white counterclockwise arrow). However, the corresponding PS appeared on the phase map only over 7% of the simulation time. Other times, the RWE was represented at the basket phase map as a wave encircling the south pole without a PS. Additionally, false PSs were also found, as follows: at splines E-F-G the basket detected a pair of imaginary PSs (IMPSs, black arrows in Fig 5A, bottom) during the entire simulation time (splines near the TV orifice), and the basket phase map pattern was differed from the phase map pattern at the endocardium-blood interface. On the other hand, at electrodes B7-B8-C8-C7, the basket phase map displayed false interpolation PSs (FIPSs) 57% of the simulation time (grey arrow in Fig 5A bottom). Fig 5B depicts a selection of points (P1, P2 and P3) at the CT rotor meandering area (top) as well as snapshots of the basket phase maps when the CT rotor tip is located at or near each of these points. As demonstrated by the three sequential basket phase maps at 1000, 1580 and 8600 ms, the CT rotor (green dots) was not detected when it moved toward the IVC, whereas the IMPSs (red dots) appeared for the entire simulation time (see also S2 Video). Fig 5C shows the EGMs at some of the electrodes detecting the CT rotor (C3, B2 and B3), a IMPSs (F4, F5 and G4), the RWE (C7, B8 and B7) and FIPSs (C8, C7 and B8). Green, red and blue arrows superimposed on the plots of the EGMs illustrate the sequential activation of the electrodes for the CT rotor, IMPSs and RWE respectively (blue circles on the EGM plot depict the activation times). However, the grey superimposed arrow shows the non-sequential activation of the electrodes in case of FIPSs. At t = 10000 ms, the basket phase map (Fig 5A, bottom) shows the RWE and a FIPS between electrodes B7, B8, C7 and C8. As demonstrated by the EGMs in Fig 5C, the counterclockwise activation corresponds to the RWE, being the FIPS an artifact due to the interpolation of the EGMs. The phases corresponding to EGMs in Fig 5C are shown in S3B Fig.

The basket phase map also differed substantially from the endocardium-blood interface phase map when the basket was set at the CT position (Fig 6). At splines A-B-C, the CT rotor was detected 90% of the simulation time. During the other 10% the CT rotor had migrated closer to the SVC from which all basket electrodes were at d > 0.5 cm (see Fig 6A). As for the RWE, its propagation was detected uninterruptedly at electrodes located at the basket’s south pole, although no corresponding PSs appeared on the phase map because the south pole (as well as the north pole) area was not interpolated (see S4 Fig). Greater discrepancies between the endocardium and basket maps are visible at splines E-F-G, since in the CT position of the basket a higher number of electrodes corresponding to such splines were located at d > 0.5 cm (see Fig 2) from the atrial surface. Moreover, an IMPS was noticed at splines E-F-G-H 100% of the simulation time. Finally, FIPSs appeared at electrodes B1-B2-C1-C2 and D4-D5-E4-E5 13% of the simulation time. Fig 6B shows that the CT rotor (green points) was not detected when it was near the SVC. In contrast the IMPS (red points) was always present (see S3 Video). Fig 6C shows the EGMs at some electrodes detecting the CT rotor (B6, C6 and C5), the IMPSs (F2, G2 and G1) and FIPSs (B1, B2 and C1). Results are in accordance with those obtained for the basket in the SVC position: the activation arrived sequentially to the electrodes in case of the CT rotor and the IMPSs, but not in case of FIPSs. Phases corresponding these EGMs are shown in S3C Fig.

Fig 7A illustrates the mapping when the basket was set at the CS position. The CT rotor was detected in this basket position only 35% of the simulation time at splines H-A-B between the first and second ring of electrodes in the basket (see Fig 7A bottom). Detection of the CT rotor was poor because the rotor meandering area was relatively far (d>1 cm) from splines A-B-C of the basket in this position (compare Fig 7A, bottom, and Fig 2). The RWE was detected at electrodes B7-C7-C8-B8 61% of the simulation time. In addition, IMPSs were located along spline B (electrodes covering the SVC orifice) during 31% of the simulation time. And also, some FIPSs were detected 40% of the simulation time at electrodes E2-E3-E4-F2-F3-F4 and A7-A8-C7-C8. As in the previous cases, when comparing the basket phase map to the map on the endocardium blood interface, there were some differences, especially at splines A-B-C. As shown in Fig 7B and S4 Video, neither the CT rotor (green points) nor the IMPSs (red points) were always detected. As in the other 2 basket’s positions, Fig 7C shows the EGMs at some of the electrodes detecting the CT rotor (H1, H2 and A1), IMPSs (B3, C3 and B4), the RWE (B7, B8 and A8) and FIPSs (B7, B8 and C8). As in those basket’s positions, activation arrived sequentially to the electrodes in case of the CT rotor, IMPSs and RWE, and there was not sequential activation of the electrodes in case of the FIPSs. The phases corresponding to the EGMs in Fig 7C are shown in S3D Fig.

The detailed analysis of basket detection for the three considered positions yielded a high detection rate (~90% of the simulation time) of the real rotor, i.e. the CT rotor, when the basket was closely covering the rotor meandering area (SVC and CT positions, as shown in Fig 4A). In the case of the basket at the CS position, detection of the CT rotor was poor due to the electrodes distance from the meandering area (see Fig 4A). However, basket-based phase maps in our simulated setting always generated a number of phantom rotors (IMPSs and FIPSs) in the presence of only one real rotor (CT rotor) for each of the basket positions. (It should be noted that the PS associated with the RWE around the IVC is not to be considered false due to the fact that multi-electrode recording systems will produce a PS for both anatomical and functional reentries.) Furthermore, after computing the instantaneous location of the CT rotor on the endocardial surface along the simulation time, as well as the location of the CT rotor detected by the basket in the three different positions studied, we calculated the distance between the real and detected trajectory of the rotor at any moment of the simulations and obtained that: i) for the SVC position this distance was between 0.37 and 1.38 cm (median 0.69 cm); ii) for the CT position it was between 0.43 and 2.48 cm (median 0.94 cm); and iii) for the CS position it was between 2.53 and 3.97 cm (median 2.88 cm). This supports the fact that rotor localization is more accurate when the basket is properly located inside the atrium in close proximity to the region of the rotor.

#### Effect of ectopic and rotor activity on IMPSs

To rule out the possibility that IMPSs result from the ectopic pacing close to the CS, the simulation was prolonged for additional 5000 ms following secession of the pacing. The activation patterns in the atria changed; they became dependent on a drifting reentry alternating between anatomical and functional cores. Nevertheless, we observed clear IMPSs on the phase maps of the basket at SVC and CT positions, as in the simulation that includes the CS pacing.

After two rotation cycles, the CT rotor became a reentry around the SVC (SVCR). Then at instant 11000 ms it migrated to the area between the BBRA and the RAA, where it kept rotating for about 20 cycles as a rotor. Later at instant 12500 ms it came back to the SVC and behaved again as a reentry circulating around the SVC until the end of the simulation. The RWE became the extension of the SVCR and kept circulating around the IVC for the whole simulation time (IVCR). Snapshots of the basket’s phase maps based on the HT of the filtered EGMs in this simulation without pacing are depicted in S5 Fig. As demonstrated in this basket phase maps, IMPSs are still generated even though the train of ectopic foci was not active. On the basket’s phase maps, we observe the SVCR (white arrow) and the IVCR (white dashed arrow) for the three basket’s positions. However, we observe IMPSs (black arrows) in the TV region just in case of SVC (panel A) and CT (panel B) positions, as occurred in the previous simulation with the CS pacing. For the CS position (panel C) the percentage of IMPSs was completely negligible. This evidence suggests that high frequency pacing shouldn’t be considered the leading cause of IMPSs generation, although it might help depending on the position of the pacing with respect to the basket.

### Effect of the far-field sources on mapping

**Fig 8:**
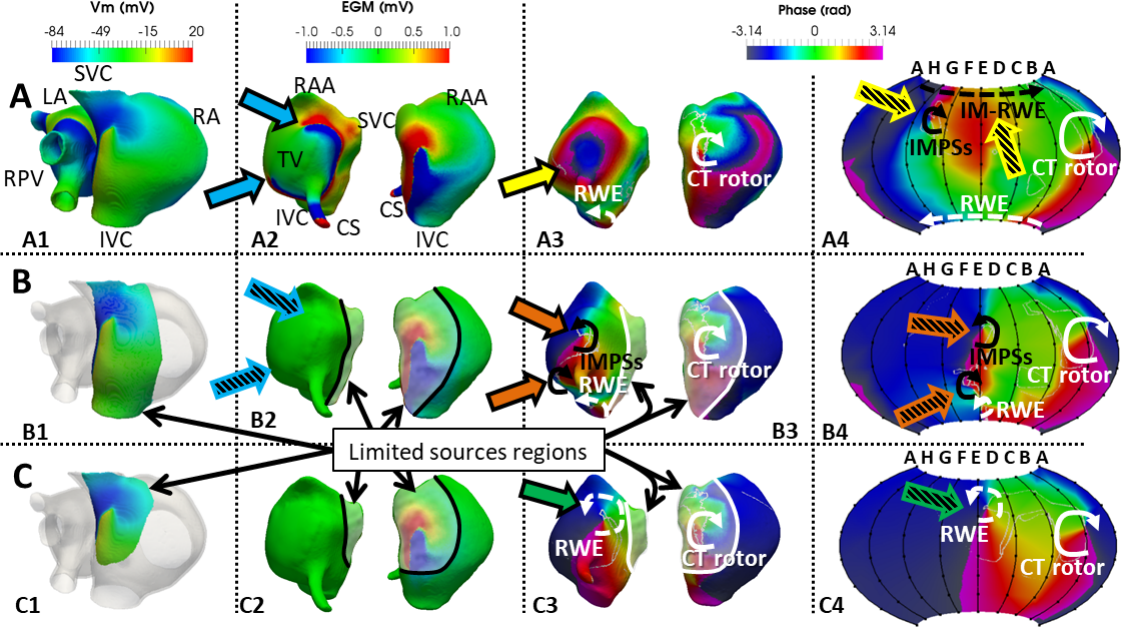
Effect of the far-field sources. Maps for the endocardial and CT positioned basket were generated for 3 different distributions of sources. All maps are shown at t = 4935 ms. A) Sources include the whole RA and LA (A1), B) sources include tissue only encompassing the CT rotor and the RWE at the IVC (B1), and C) sources include only tissue encompassing the CT rotor (C1). A2, B2 and C2: Voltage maps of the EGMs computed on the endocardium-blood interface corresponding to the whole (A2) and limited atrial sources (B2 and C2). Blue arrows (A2) indicate edges of EGM wave propagating around the TV. Hatched blue arrows in B2 indicate that once the sources are removed from the TV region, no significant voltage gradients are visible in that location. A3, B3 and C3: Phase maps computed on the EGMs maps from the endocardium-blood interface. In the whole sources map (A1), no PS is visible at the moment of this analysis (yellow arrow indicates a short living PS at earlier time). Orange arrows (B3) indicate PS outside of the sources region which is therefore an IMPS resulting from far field sources. Green arrow (C3) indicates a dramatic shift of the RWE into a region outside of the sources region. A4, B4 and C4: Phase maps from the recordings of the virtual basket. Hatched yellow arrows (A4) show IMPS in the region of the TV and an imaginary rotor wave extension (IM-RWE) around the basket north-pole (see text). Hatched orange arrows (B4) indicate the IMPS on the basket phase map. C4 is the basket phase map with limited sources shown in C1. Hatched green arrow indicates a PS corresponding to the RWE, but with a dramatic shift in location relative to A4. White and black lines on B2-3 and C2-3 depict the limits of the sources region. CT rotor: rotor along the crista terminalis; RWE: rotor wave extension; IMPS: imaginary phase singularity; IM-RWE: Imaginary RWE; LA and RA: left and right atrium; RPV: right pulmonary veins; SCV and IVC: superior and inferior vena cava; RAA: right atrial appendage; CS: coronary sinus; TV: tricuspid valve.

To verify the importance of far-field sources in the genesis of IMPSs, we computed the endocardial and basket maps when considering activity generated by decreasing areas of endocardial wall activity. As an example, Fig 8 shows results at t = 4935 ms for the CT position of the basket. The left most column displays the 3 spatial extensions of AP sources considered in the analysis: A1 includes the entire atrial tissue, B1 includes only tissue encompassing the CT rotor and its RWE, and C1 considers only tissue closely encompassing the CT rotor (see S6 Fig); A2, B2 and C2 display endocardial-blood interface EGM; A3, B3 and C3 display endocardial-blood phase maps; and A4, B4 and C4 display the basket phase maps.

When considering the whole atrial tissue (A1), the CT rotor (white curved arrow) and the RWE (dashed white curved arrow) were detected in both the endocardium-blood interface (A3) and the basket phase maps (A4). Additionally, an IMPS and an imaginary extension of that rotor (IM-RWE, hatched yellow arrows) appeared on the basket phase map in an area close to where the endocardial-blood EGMs showed propagation around the tricuspid valve (TV) annulus (blue arrows in A2). As the IMPS was not detected on the endocardium-blood interface phase map (A3) but was detected on the basket mapping (A4) we further investigated whether IMPS are formed by far-field signals by excluding the nearby sources. When considering the sources as the activity at the CT rotor and RWE alone (B1), the EGMs in the TV area show very small voltage amplitude (hatched blue arrows in B2) that gives rise to IMPSs on the endocardium-blood surface (orange arrows in B3), as well as on the basket phase maps (hatched orange arrows in B4). These orange arrows (B3) indicate PSs outside of the sources region; that is, they are IMPS resulting from far field sources (see also S6 Fig). A magnification of the amplitude scale of the EGMs reveals that the IMPSs in this case arise from the high sensitivity of the phase analysis to low amplitude waves far from the sources (see S5 Video and S7 Fig). Finally, phase maps of sources confined to only close vicinity of the CT rotor core (C1) removed the IMPSs and detected the CT rotor and its extension reentry toward the boundary of the source region (C3-C4), also termed RWE. The PS corresponding to the CT rotor remained close to its original location, shown in panels A3 and A4. However, the RWE suffered a dramatic shift in location relative to its origin, shown in panels A3-A4, and resided outside of the active sources region.

The data in Fig 8 demonstrate that basket IMPSs were a consequence of the distal atrial tissue activation at either the CT rotor and the IVC RWE, or at the TV region. In the example provided, far-field sources interfered with the recordings when electrodes were at distances greater than about 0.5 cm from the endocardial wall activity. The effect was observed in the basket phase map when considering the whole atrial tissue (Fig 8A) and on the endocardium-blood interface and the basket phase maps when considering the tissue encompassing the CT rotor and its RWE (Fig 8B). But if distance keeps increasing or source shrinking, the influence of far-field sources is not enough to generate IMPSs, as demonstrated by considering only the tissue encompassing the CT rotor (Fig 8C).

### Effect of the inter-electrode interpolation on mapping

**Fig 9:**
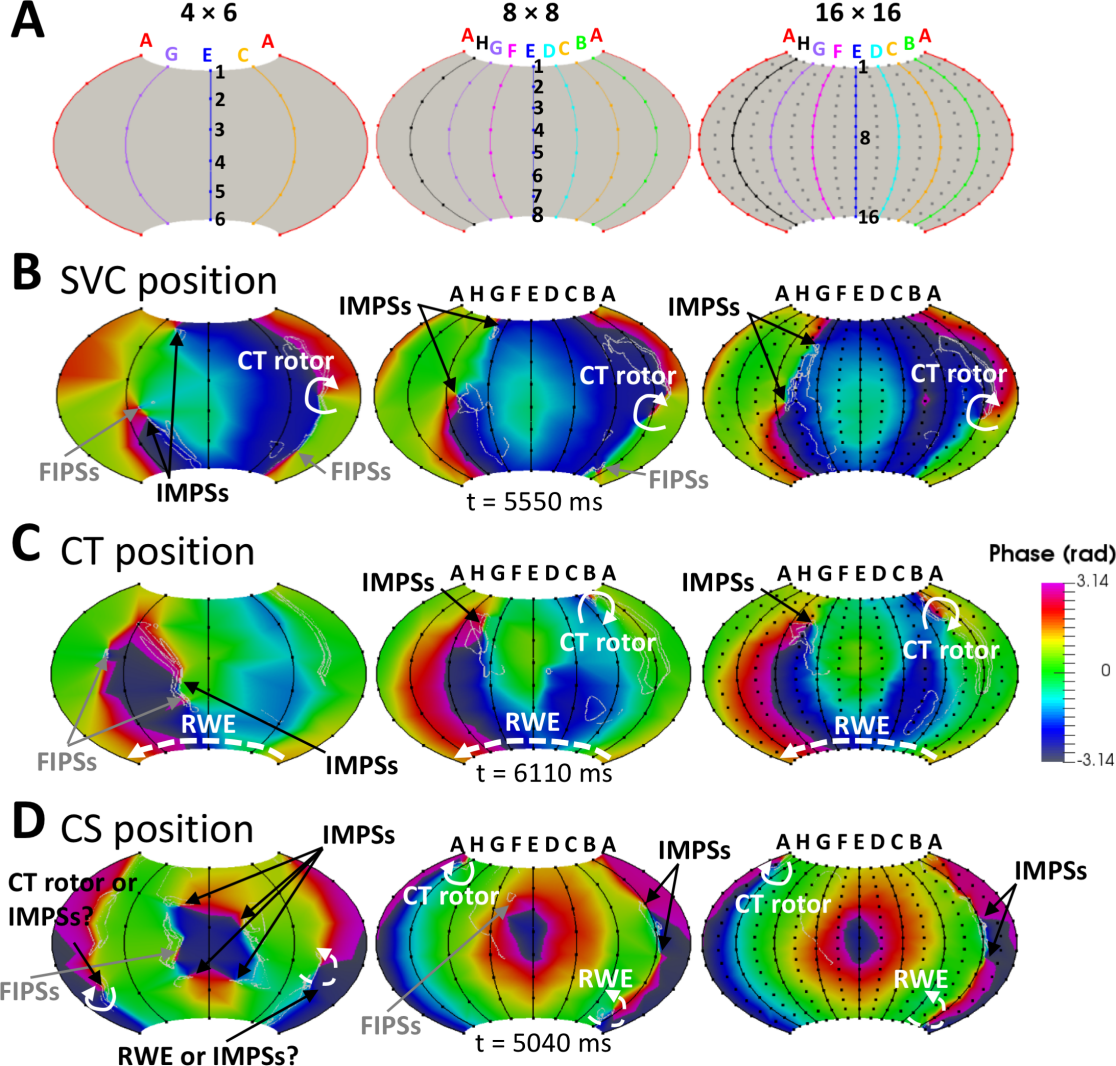
Effect of EGMs interpolation in the phase maps. 2D projection of the baskets (A) and phase maps for the SVC (B), CT (C) and CS (D) positions with a density of electrodes of 4×6 (left), 8×8 (middle) and 16×16 (right). IMPSs: imaginary phase singularities; FIPSs: false interpolation phase singularities; CT rotor: rotor along the crista terminalis; RWE: rotor wave extension.

We hypothesized that reducing the inter-electrode distance, would reduce the percentage of FIPSs. Therefore, we analyzed the effect of modifying the number of electrodes in the basket by decreasing it to 4×6 or increasing it to 16×16 (Fig 9A). Fig 9B–9D are snapshots of the basket phase maps for each of the three positions (see also S1 Table). Increasing the electrodes density at the SVC position improved the ability to detect the CT rotor from 85% (4×6) to 94% (8×8) and 97% (16×16) of the time; at the CT position from 46% (4×6) to 90% (8×8) and 94% (16×16); and at the CS position from 35% (4×6 and 8×8) to 63% (16×16). Clearly, improvement was less in the CS position because the basket was located farthest from the CT rotor meandering area. Notably, at an electrode density of 4×6 in either the SVC or CS position, the detection of the RWE was impaired by the appearance of the FIPSs. Increasing the electrode density from 8×8 to 16×16, improved detection slightly (7 to 9%) for the SVC position, but remained unchanged for the CS position (61%). Whereas in the SVC and CT positions IMPSs were present 100% of the simulation time regardless of the electrode density, the percentage of time during which FIPSs were present went down considerably (from 70% to 57% and 0% for the SVC position and from 59% to 13% and 8% for the CT position) when increasing the electrode density. In the CS position, in addition to a poor detection of the CT rotor, the 4×6 electrode basket yielded an extremely high percentage of FIPSs and IMPSs during the entire simulation time and it was almost impossible to differentiate between them. Increasing the density of electrodes allowed differentiating IMPSs from FIPSs, so the FIPSs percentage was reduced to 40% for 8×8 electrodes and eliminated for the 16×16 electrode basket density.

In addition, we computed the phase maps with ~900 interpolated points for the 8×8 basket, and as expected, the number of FIPSs was lower than for the phase maps with 57600 interpolated points. This fact confirmed our hypothesis regarding the effect of increasing the resolution of the phase mapping through increasing the number of interpolated points, which yields a higher number of FIPSs. This effect was also shown when comparing the 8×8 basket with the 16×16 basket: both had 57600 interpolated points but in case of the 8x8 basket the number of interpolated points doubled and the number of FIPSs was higher.

To sum up, CT rotor detection accuracy improved slightly when increasing the number of electrodes above 8×8 for the SVC and CT positions. Improvement was significant for the CS position, in which the rotor coverage was poor. In addition, no FIPSs appeared for the SVC and CS positions, whereas the percentage of FIPSs decreased slightly for the CT position. However, accuracy worsened considerably and the percentage of FIPSs increased greatly when decreasing the number of electrodes. Finally, the percentage of IMPSs remained stable regardless of the electrode density.

### Discussion

To date the accuracy of mapping AF to localize rotors using panoramic basket catheters has not been validated, in part because in clinical practice the fibrillatory activation patterns are not known. We have used computer simulations to analyze in detail factors affecting AF mapping and localization of rotors. Our results show that rotors may be identified by phase maps of electrical recordings directly from the endocardial surface-blood interface, but less reliable so by phase maps built from basket catheter recordings. Importantly, a potential inaccuracy of the basket maps includes phantom rotors, which may confound targeting of ablation to terminate AF. Our analysis suggests that the appearance of the phantom rotors can be attributed to at least three factors: The distance between the basket electrodes and the endocardial wall (positioning), the distance between the atrial waves and the electrodes (far-field) and the inter-electrodes distance (interpolation) of data used to create the maps. Therefore, our results suggest that while phase maps based on basket catheters are a powerful tool to map AF and to localize real rotors and other ablative targets, they can also mislead physicians to ablate atrial regions that are in fact free of rotor sources of AF.

### Effect of the electrode-endocardium distance

The distance between the endocardial wall and the basket electrodes depends on the basket´s position within the atria. At each electrode location distance changes non-uniformly, which clearly affects rotor detection. Our results reveal that in addition to the amount of RA area coverage with small distance (d≤0.5cm), coverage of the rotor meandering area is important. In our simulations, the basket at the CS position was the farthest from the rotor meandering area and therefore it detected the rotor only 30% of the time, which was much lower than the SVC and CT positions at which the basket was closer to the rotor area and detection was over 90% of the simulation time. On the other hand, the false rotors (IMPSs and FIPSs) tend to appear in basket regions with d>0.5 cm.

Thus, basket positioning with gaps between the electrodes and the endocardium may lead to poor rotor detection and probably low rates of AF termination[18,19,61,62]. Our data agree with results by Narayan et al[15], in which ablation of drivers slowed but did not terminated AF when the atrial coverage was poor because of the limited size of commercial baskets compared to the large size of the atrium. Narayan et al attributed the failure to the existence of residual sources in the unmapped regions. However, according to our results, unsuccessful AF termination may have been also due to ablation of phantom rotors appearing at electrodes near and distant from the true rotors locus. Unfortunately, according to our simulations, false detection of rotors cannot be excluded, because even short electrode-to-endocardial wall distances do not guarantee the elimination of false rotors. We found that the endocardial-blood interface maps could show false rotors as well, likely because of far-field contribution of sources and the high sensitivity of the phase mapping to low amplitude signals (Fig 8). Interestingly, when the distance between the basket electrodes and the endocardium increases (after excluding part of the atrial tissue in the computations), IMPSs tend to disappear (Fig 8C), probably because scroll wave filaments originating at endocardial IMPSs do not reach deep into the cavity[52].

### Effect of basket catheter electrode density

Our results confirm that an 8×8-pole mapping basket catheter can yield sufficient spatial resolution for rotor detection when it is properly located in contact with the tissue at the rotor meandering area (SVC and CT positions in our simulations). Increasing the electrode density did not significantly improve rotor detection. We however predict that decreasing the electrode density (e.g., 4×6) from an optimal level will reduce the ability to detect rotors, while increasing it (e.g., 16×16) will not substantially alter results if the basket is located close to the rotor area (a substantial improvement was observed only for the basket at the CS position, where the basket did not cover the meandering area of the rotor).

Other studies are consistent with our observations. Narayan et al[8] showed that irregular inter-electrodes distances do not alter the sequential activation across adjacent electrodes surrounding a rotor. In addition, the study by Rappel and Narayan[16] suggested that the spatial resolution of a 64-pole mapping basket catheter is adequate to detect rotors, although noise in the EGMs and electrode position might affect accuracy. This is in accordance with our results showing that CT rotor detection was good for a 64-pole basket positioned in the SVC and CT, whereas it was poor for the CS position.

Recently Roney et al[63] found that basket catheters are prone to false detections and may incorrectly reveal rotors that are not present, and also that increasing the number of splines up to 16 reduces both the number of false PSs and the number of missing PSs. In general, our results are in accordance with their results. When we increased the number of splines up to 16, for the three basket positions the false PSs due to the interpolation (FIPSs) were strongly reduced and the sensitivity for detection of the real rotor increased (see S1 Table in the supplemental material). However, our study highlights the fact that rotor tracking is more effective if the basket catheter is placed appropriately inside the atrial cavity to ensure extensive coverage of the rotor meandering area. Indeed, false rotors appearing as a result of a larger than critical electrode-to-tissue distance (i.e., IMPSs) will persist even after improving the spatial resolution (Fig 9). Furthermore, for certain positions of the basket, the rotor would not be detected if it drifts to a poorly covered region, as seen for the SVC position when the rotor migrated toward the IVC and for the CT position when the rotor migrated toward the SVC (see Fig 4).

It should be noted that, in the clinic, if the basket is not large enough, it would not be possible to determine if it is properly located inside a cavity because one would not know a priori the rotors’ location. Averaging all three positions analyzed, detection of the CT rotor with the 8×8 basket occurred 73% of the time, whereas with the 16×16 basket it was detected 85% of the time, which suggests some improvement with the added electrodes. IMPSs also seem to be detected by the basket at all densities, mostly in electrodes separated from the endocardium by >0.5 cm. Averaging all three basket positions, IMPSs are detected 77% and 81% of the time with the 8×8 and 16×16 basket, respectively. On the other hand, false rotors due to interpolation (i.e., FIPSs) appear only when inter-electrode distances are large (reducing the inter-electrode distance by increasing the density to 16×16 substantially decreased, or eliminated, the occurrence of FIPSs). Averaging all three positions, FIPSs are detected 37% and 4% of the time with the 8×8 and 16×16 basket, respectively. Overall, our simulations suggest that the probability of ablating an erroneous target would be higher than the probability of correctly ablating a target when using a small 8×8 basket to guide ablation. However, the probability of correctly ablating a target would increase substantially when using a small 16×16 basket.

### Limitations

We need to consider several potential limitations of our study. The 3D atrial model is anatomically and electrophysiologically realistic, but is simplistic regarding wall thickness and ionic heterogeneous details. Although it does not alter our main conclusion, the preferable distance to avoid imaginary rotors (IMPSs; < 0.5 cm in our simulations) could be dependent on the anatomy of the atrial model; for example, it is likely that if the atrial wall thickness would change, this distance would also change. In addition, we have presented simulations for a single scenario of a relatively large rotor area without additional wavebreaks. Such considerations limit our ability to extrapolate quantitatively the results to other fibrillatory wave propagation scenarios. In addition, our study used a single signal processing protocol that included band-pass filtering and the Hilbert Transform, together with an automatic PS detection. We did not explore other EGM processing methods that could have affected the rotors detection, however the protocol used is considered generic to phase mapping and as such very clinically relevant. Another factor limiting the accuracy of basket-based phase maps is the quality of the signals used here compared to the actual clinical EGM signals, which are usually contaminated by far-field effects from the ventricles and noise. Patterns of real atrial waves and signals during AF are probably more complex and with a higher number of artefacts than those simulated here, which could decrease the reliability of the computed phase maps. Finally, we simulated the EGMs recorded by spherical mapping basket catheter located inside the RA and no deformations were applied. Although somewhat unrealistic, this geometrical configuration is well suited to highlight the clinically important effect of varying the distance between the electrodes and the endocardium[15,18,19] as well as the difference between the basket phase maps and the endocardium-blood interface phase maps. Moreover, our characterization of the various effects of specific basket positions on detection of true and false rotors is based on solid theoretical principles that are commonly accepted in cardiac electrophysiology and provide insights into the drawbacks of using basket catheter-based phase mapping of AF rotor sources.

### Conclusions

We demonstrate that atrial rotor detection can be achieved by a phase analysis of multi-electrode basket catheter positioned at any distance from the atrial tissue, but preferably placed closer than 0.5 cm to the atrial tissue as basket electrodes far from the tissue tend to produce false rotors in addition to real rotors due to the increased effect of distant activity. We further demonstrate that distant activity can also produce imaginary PSs in phase maps even at short electrode-to-endocardial wall distances and without interpolation. Overall, maintaining the basket electrode grid at 8×8 or higher seems sensitive enough for detection of large area rotors, although accuracy will vary depending on the position of the basket inside the atrial cavity and the number of electrodes. Importantly, although basket catheters are currently used to guide patient-specific ablation of the AF drivers, spurious targets in the form of phantom rotors cannot be excluded and all detected rotors should be cautiously considered.

## Supporting information

S1 Fig Atrial activation times. Snapshots of the atrial activation spread following Sino-atrial node (SAN) activation and comparison of the simulated activation times at different points of the atria with the experimental activation times reported by Lemery et al[36].
(TIFF)

S2 Fig Inter-electrode distances. Inter-electrode distances for 4 x 16 (A), 8 x 8 (B) and 16 x 16 (C) basket configurations.
(TIF)

S3 Fig Phases computed based on the HT of filtered EGMs. Plots of the phases: in the endocardium corresponding to points 1–5 (left) and 6–10 (right) in Fig 3 (A); at some electrodes in the basket for the SVC (B), CT (C) and CS (D) positions, corresponding to Fig 5–7 respectively.
(TIF)

S4 Fig Phases at the basket’s south pole. A) Phases in the last (south-most) ring of electrodes (A8 to H8) at t = 5200 ms when the basket was located at the SVC, CT and CS positions. Only in case of the CT position a circulating activation corresponding to the RWE (black arrow) can be observed, as shown by the phases’ color code. In case of the SVC and CS positions we are detecting the collision between the CS stimuli wavefront and the RWE and we are not observing the RWE due to the lack of electrodes at the basket’s south pole. B) Plot of the phases in the last ring of electrodes (SVC position: top; CT position: middle; CS position: bottom). SVC and IVC: superior and inferior vena cava; LA and RA: left and right atrium; RPV: right pulmonary vein; TV: tricuspid valve area.
(TIFF)

S5 Fig Basket phase maps of simulation following secession of the high frequency pacing close to the CS. Snapshots for the SVC (A), CT (B) and CS (C) baskets positions are shown for 11200 and 15000 ms time points and demonstrate IMPSs presence as in the simulation that includes the stimuli train. SVC and IVC: superior and inferior vena cava; TV: tricuspid valve; SVCR and IVCR: reentry around the SVC and IVC; IMPSs: imaginary phase singularities; FIPSs: false interpolation phase singularities.
(TIFF)

S6 Fig Sources regions to analyze the effect of far field. A) Views of the tissue encompassing the CT rotor and the RWE (A1), IMPSs and RWE (A2) and CT rotor (A3) on the endocardial phase maps. B) Views of the tissue encompassing only the CT rotor (B1), RWE (B2) and CT rotor (B3) on the endocardial phase maps. Black lines enhance the border of the sources region overlapped to the endocardial phase maps.
(TIF)

S7 Fig Sensitivity of the phase maps. Color scale magnification in the EGMs maps shown in Fig 8 (first and second column), and corresponding traces of the EGMs at points 1 and 2 when considering for the computations with the limited sources regions as shown in Fig 8: whole atrial tissue (A1), tissue encompassing the CT rotor and the RWE (B1) and tissue encompassing the CT rotor (C1). Point 1 is located on the TV area, while point 2 is on the CT rotor meandering area.
(TIFF)

S1 Video Simulated atrial propagation and phase maps on the endocardium-blood interface. A) Electrical propagation in the atria along the simulation time; B) Phase maps on the endocardium-blood interface, whose computation was based on the HT of the filtered EGMs. PS detections are overlapped to the phase maps (white).
(AVI)

S2 Video Basket’s phase maps in the SVC position. Color-coded phase maps computed based on the HT of the filtered and interpolated basket’s EGMs. PSs are depicted in white superimposed over the phase maps.
(AVI)

S3 Video Basket’s phase maps in the CT position. The layout of this video is the same as in S2 Video.
(AVI)

S4 Video Basket’s phase maps in the CS position. The layout of this video is the same as in S2 Video.
(AVI)

S5 Video Phase maps hypersensitivity. Video of the EGMs and phase map corresponding to Fig 8 and S7 Fig.
(MP4)

S1 Table PS detections with baskets with various densities of electrodes. Percentage time of detection of the CT rotor, RWE, IMPSs and FIPSs for the 3 positions of the basket when the density of electrodes was 4×6, 8×8 and 16×16.
^a^No PSs but sequential activation A8→H8.
^b^Detection possibly masked by the FIPSs at electrodes A4-A5-A6-B4-B5-B6.
^c^The low density of electrodes renders a poor detection through the whole simulation period, including IMPSs and FIPSs.
(DOCX)
