# Supplementary figures and images for: Factors affecting basket catheter detection of real and phantom rotors in the atria: A computational study
Source: PLoS Comput Biol. 2018 Mar 5;14(3):e1006017. doi: 10.1371/journal.pcbi.1006017 (PMC5854439; doi:10.1371/journal.pcbi.1006017)

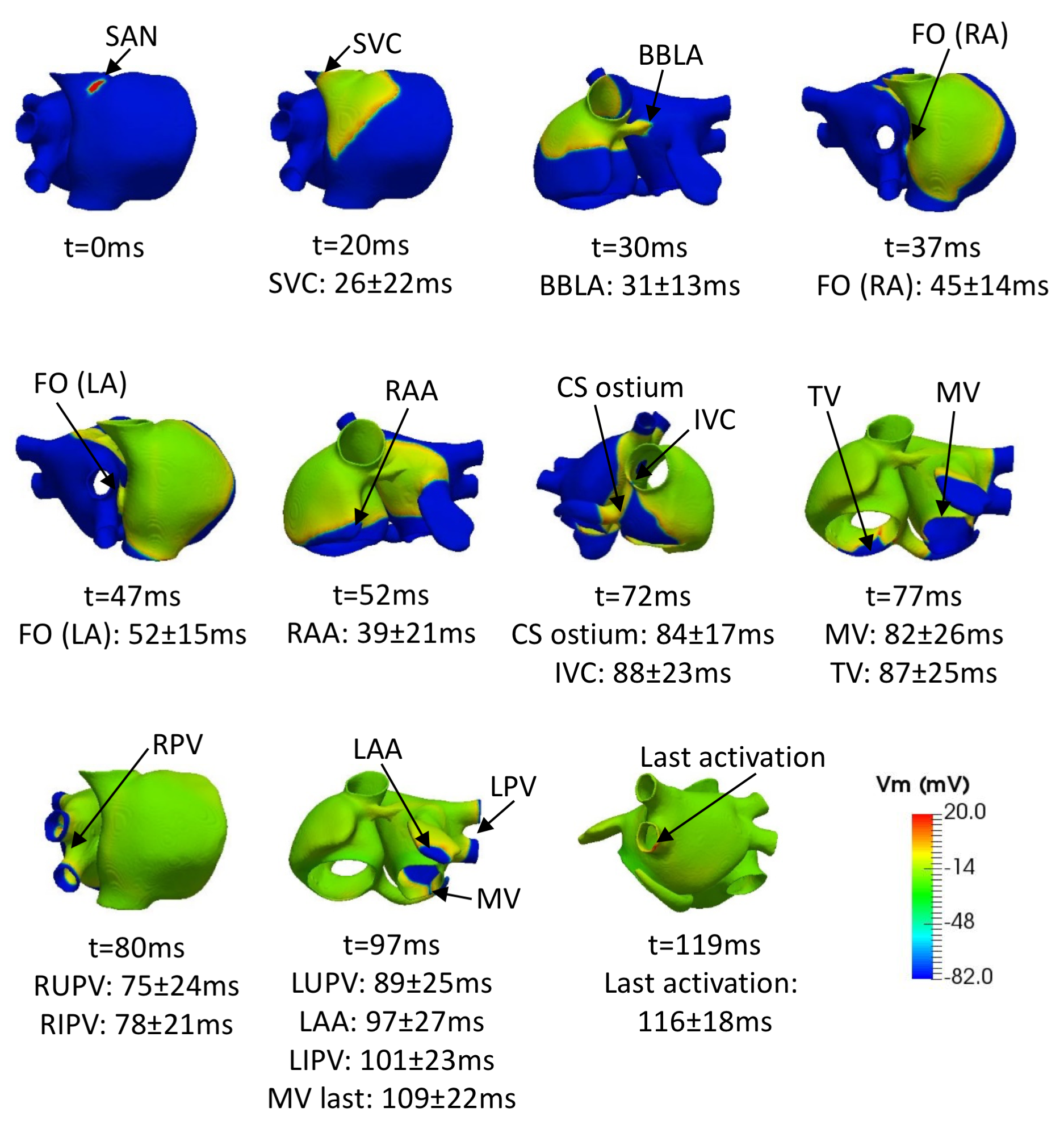

Supplement: S1 Fig — Snapshots of the atrial activation spread following Sino-atrial node (SAN) activation and comparison of the simulated activation times at different points of the atria with the experimental activation times reported by Lemery et al[36]. (TIFF) [file pcbi.1006017.s001.tiff]

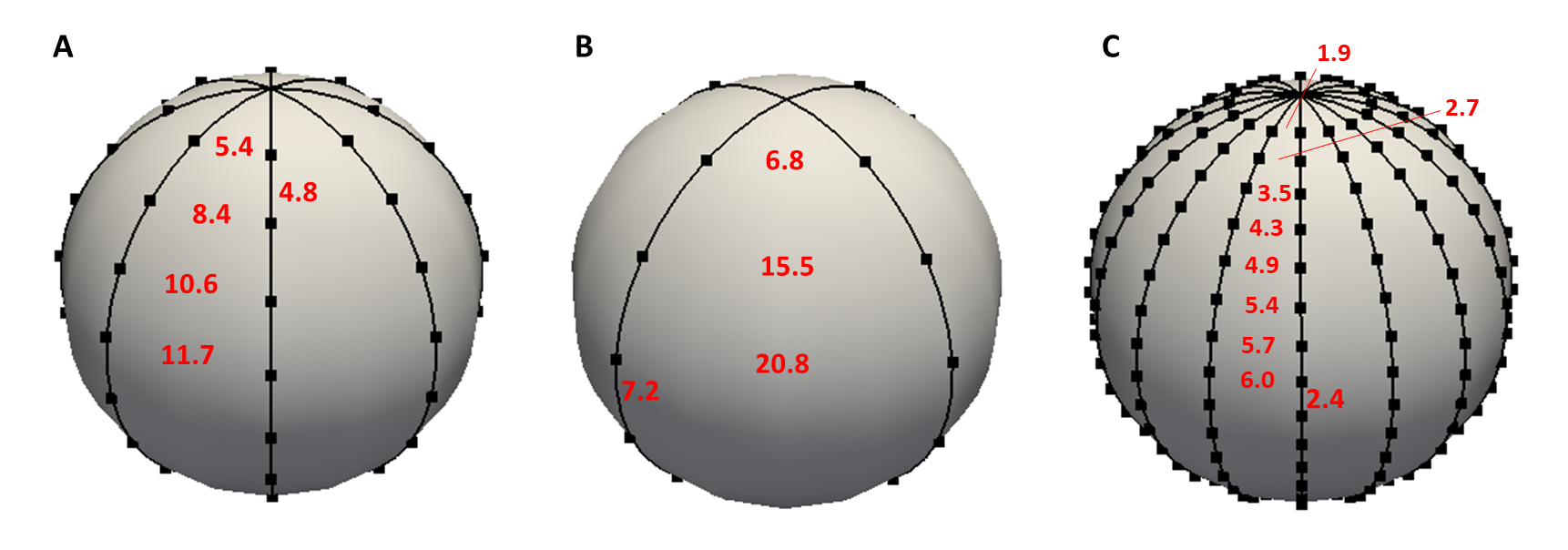

Supplement: S2 Fig — Inter-electrode distances for 4 x 16 (A), 8 x 8 (B) and 16 x 16 (C) basket configurations. (TIF) [file pcbi.1006017.s002.tif]

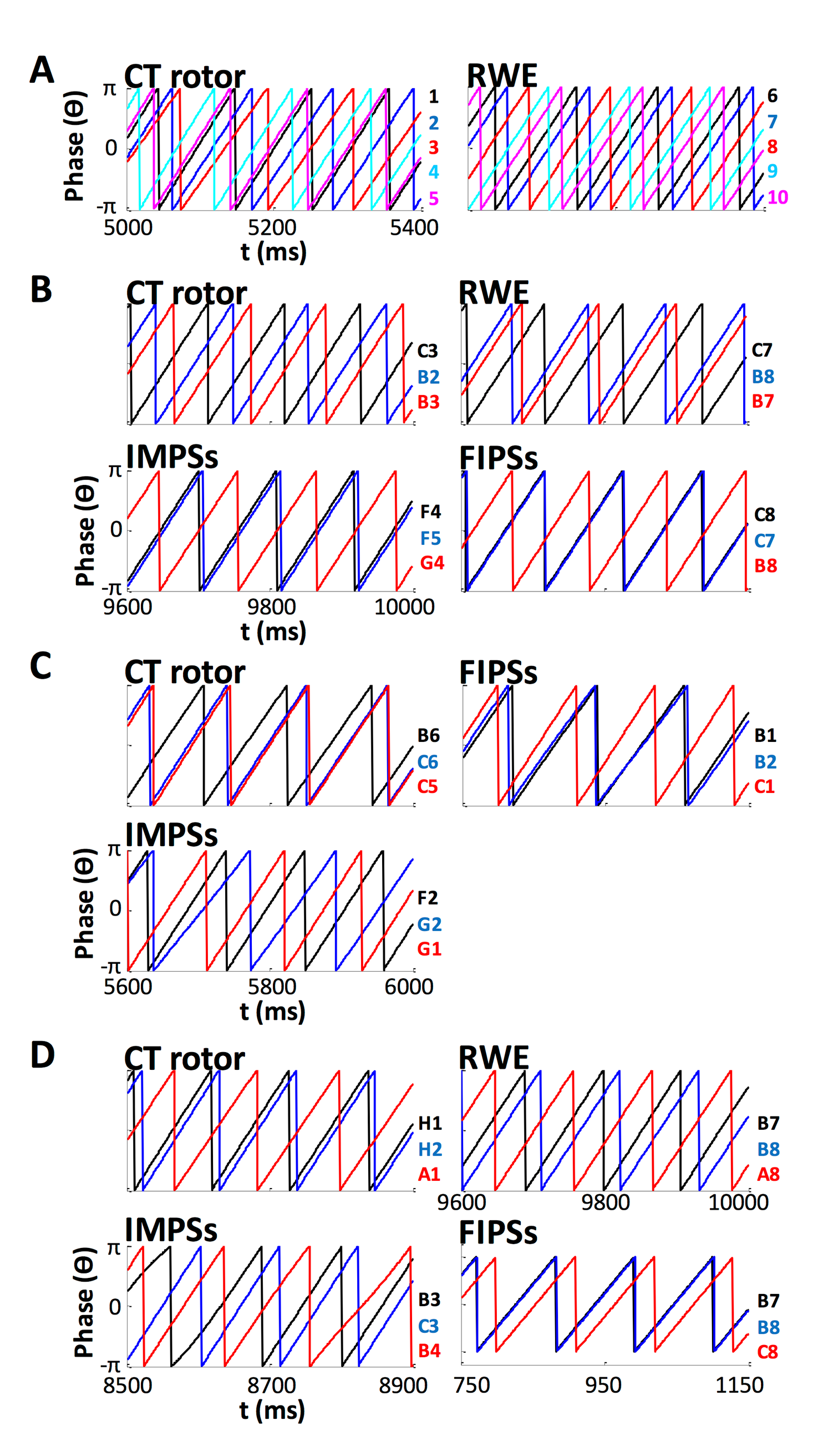

Supplement: S3 Fig — Plots of the phases: in the endocardium corresponding to points 1–5 (left) and 6–10 (right) in Fig 3 (A); at some electrodes in the basket for the SVC (B), CT (C) and CS (D) positions, corresponding to Fig 5–7 respectively. (TIF) [file pcbi.1006017.s003.tif]

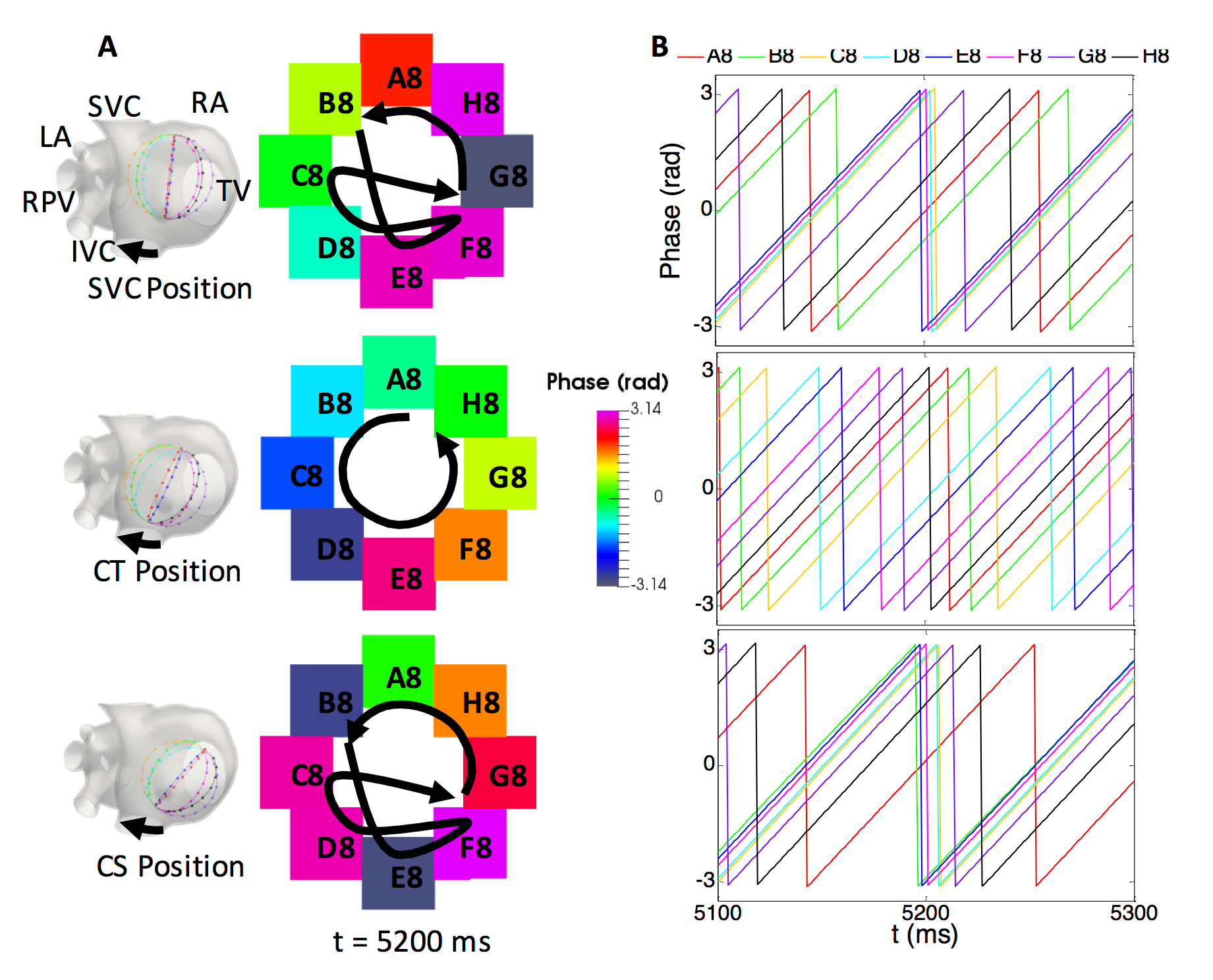

Supplement: S4 Fig — A) Phases in the last (south-most) ring of electrodes (A8 to H8) at t = 5200 ms when the basket was located at the SVC, CT and CS positions. Only in case of the CT position a circulating activation corresponding to the RWE (black arrow) can be observed, as shown by the phases’ color code. In case of the SVC and CS positions we are detecting the collision between the CS stimuli wavefront and the RWE and we are not observing the RWE due to the lack of electrodes at the basket’s south pole. B) Plot of the phases in the last ring of electrodes (SVC position: top; CT position: middle; CS position: bottom). SVC and IVC: superior and inferior vena cava; LA and RA: left and right atrium; RPV: right pulmonary vein; TV: tricuspid valve area. (TIFF) [file pcbi.1006017.s004.tiff]

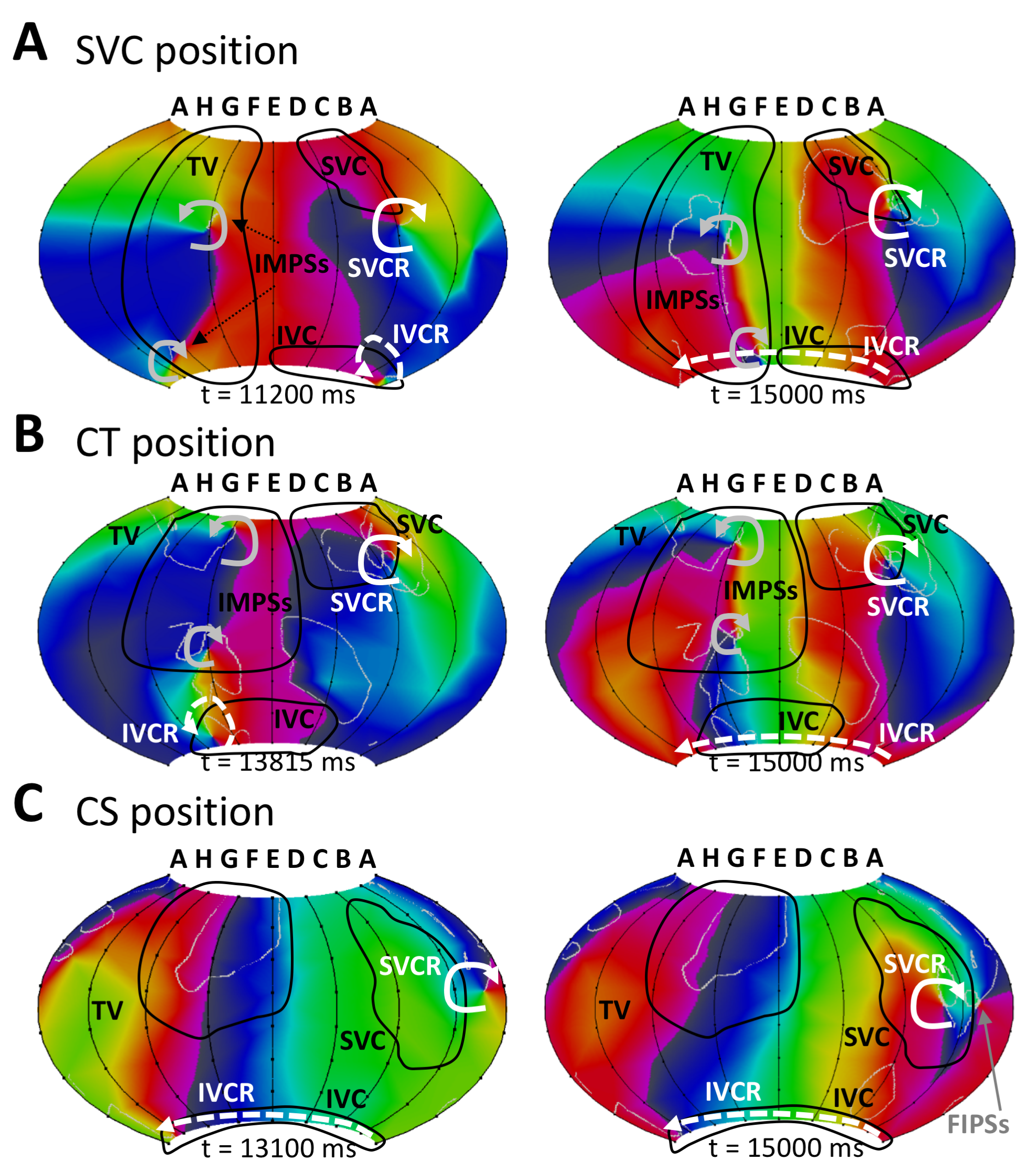

Supplement: S5 Fig — Snapshots for the SVC (A), CT (B) and CS (C) baskets positions are shown for 11200 and 15000 ms time points and demonstrate IMPSs presence as in the simulation that includes the stimuli train. SVC and IVC: superior and inferior vena cava; TV: tricuspid valve; SVCR and IVCR: reentry around the SVC and IVC; IMPSs: imaginary phase singularities; FIPSs: false interpolation phase singularities. (TIFF) [file pcbi.1006017.s005.tiff]

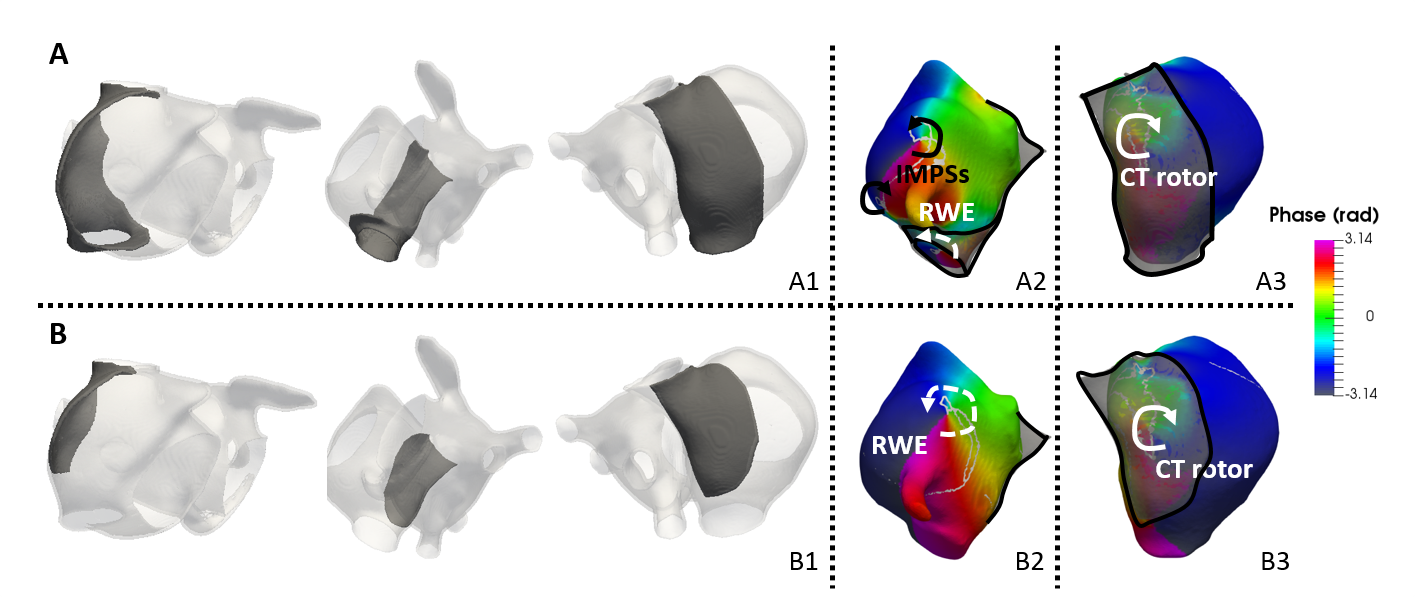

Supplement: S6 Fig — A) Views of the tissue encompassing the CT rotor and the RWE (A1), IMPSs and RWE (A2) and CT rotor (A3) on the endocardial phase maps. B) Views of the tissue encompassing only the CT rotor (B1), RWE (B2) and CT rotor (B3) on the endocardial phase maps. Black lines enhance the border of the sources region overlapped to the endocardial phase maps. (TIF) [file pcbi.1006017.s006.tif]

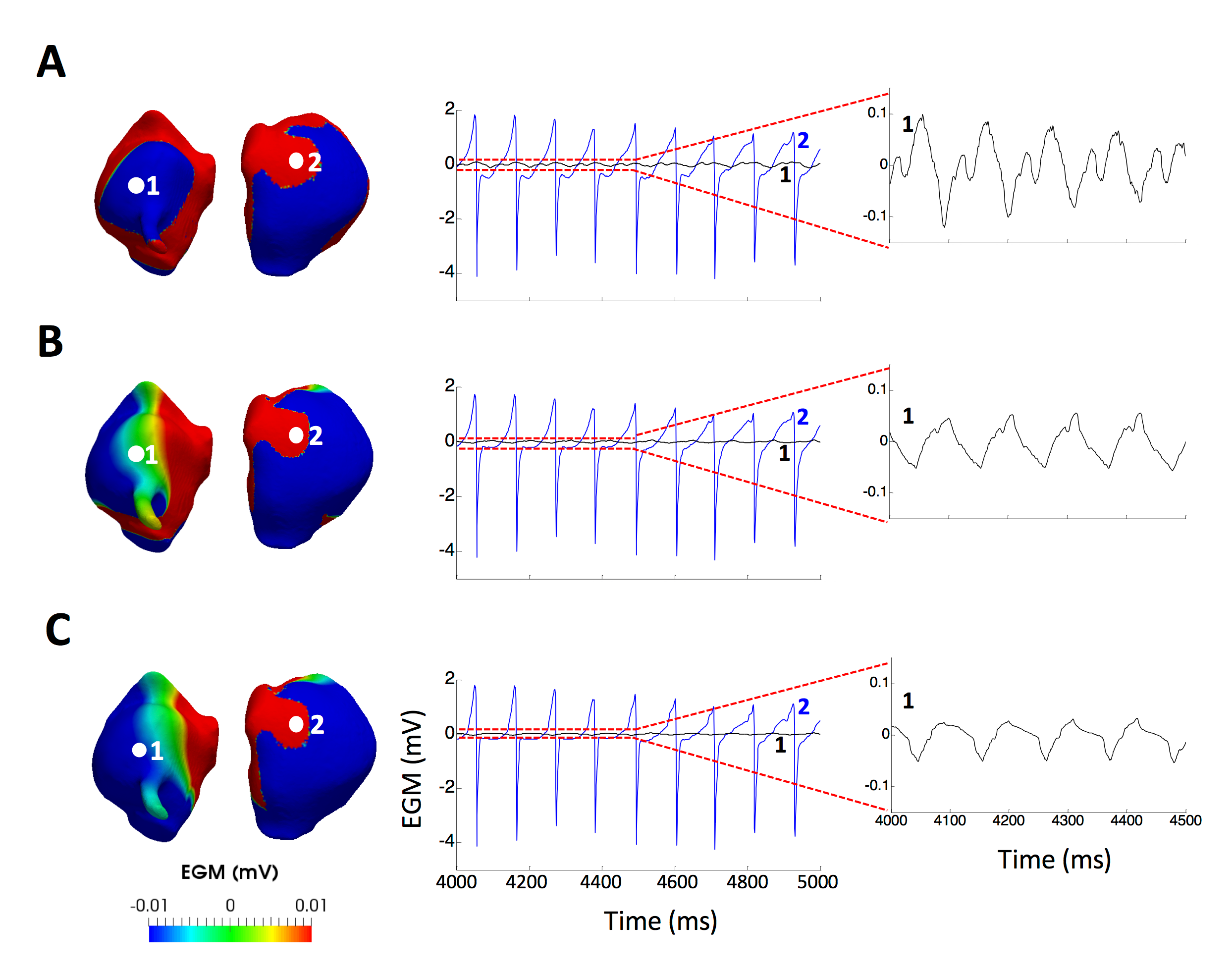

Supplement: S7 Fig — Color scale magnification in the EGMs maps shown in Fig 8 (first and second column), and corresponding traces of the EGMs at points 1 and 2 when considering for the computations with the limited sources regions as shown in Fig 8: whole atrial tissue (A1), tissue encompassing the CT rotor and the RWE (B1) and tissue encompassing the CT rotor (C1). Point 1 is located on the TV area, while point 2 is on the CT rotor meandering area. (TIFF) [file pcbi.1006017.s007.tiff]
