# Supplementary material for: Factors affecting basket catheter detection of real and phantom rotors in the atria: A computational study
Source: PLoS Comput Biol. 2018 Mar 5;14(3):e1006017. doi: 10.1371/journal.pcbi.1006017 (PMC5854439; doi:10.1371/journal.pcbi.1006017)
Supplement: S1 Table — Percentage time of detection of the CT rotor, RWE, IMPSs and FIPSs for the 3 positions of the basket when the density of electrodes was 4×6, 8×8 and 16×16. aNo PSs but sequential activation A8→H8. bDetection possibly masked by the FIPSs at electrodes A4-A5-A6-B4-B5-B6. cThe low density of electrodes renders a poor detection through the whole simulation period, including IMPSs and FIPSs. (DOCX) [file pcbi.1006017.s013.docx]

|  | SVC position | | | CT position | | | CS position | | |
| --- | --- | --- | --- | --- | --- | --- | --- | --- | --- |
| Electrode grid | 4×6 | 8×8 | 16×16 | 4×6 | 8×8 | 16×16 | 4×6 | 8×8 | 16×16 |
| CT rotor | 85 | 94 | 97 | 46 | 90 | 94 | 35 | 35 | 63 |
| RWE | 0^b^ | 7 | 9 | 100^a^ | 100^a^ | 100^a^ | 0^b^ | 61 | 61 |
| IMPSs | 100 | 100 | 100 | 100 | 100 | 100 | ^c^ | 31 | 44 |
| FIPSs | 70 | 57 | 0 | 59 | 13 | 8 | ^c^ | 40 | 0 |
